# Mid-infrared long-range surface polaritons in two-dimensional van der Waals materials

**DOI:** 10.1126/sciadv.aed2198

**Published:** 2026-08-05

**Authors:** Mingsong Wang, Shixiong Yin, Saroj Chand, Jiamin Quan, Xuefeng Jiang, Kenji Watanabe, Takashi Taniguchi, Gabriele Grosso, Andrea Alù

**Affiliations:** ^1^ Photonics Initiative, Advanced Science Research Center, City University of New York, New York, NY 10031, USA.; ^2^ Department of Electric Engineering, City College of City University of New York, New York, NY 10031, USA.; ^3^ Research Center for Electronic and Optical Materials, National Institute for Materials Science, 1-1 Namiki, Tsukuba 305-0044, Japan.; ^4^ Research Center for Materials Nanoarchitectonics, National Institute for Materials Science, 1-1 Namiki, Tsukuba 305-0044, Japan.; ^5^ Physics Program, Graduate Center, City University of New York, New York, NY 10016, USA.

## Abstract

The discovery of graphene plasmons (GPs) and hyperbolic phonon polaritons (HPhPs) in two-dimensional (2D) van der Waals (vdW) materials has enabled extreme light confinement and enhanced light-matter interactions, holding the promise for miniaturized mid-infrared (mid-IR) photonic devices. However, GPs and HPhPs suffer from limited propagation lengths, hindering their impact in various applications. Here, we demonstrate long-range surface polaritons (LRSPs) in 2D vdW materials, featuring much longer propagation lengths and faster group velocities, which may offer complementary opportunities for high-speed on-chip photonic applications in the mid-IR regime. The demonstrated LRSPs are supported by deep-subwavelength heterostructures consisting of a hexagonal boron nitride (h-BN) flake, a high-index germanium (Ge) layer, and a gold (Au) film. Within such geometry, we experimentally demonstrate propagation distances exceeding 80 micrometers crossing an entire h-BN flake without substantial decay. A theoretical prediction of ∼925-micrometer propagation length is obtained. These polaritons may be ideally suited for realizing polaritonic interconnects that bridge different components of compact and planar photonic systems, enabling mid-IR information transport and seamless integration with other vdW material–based mid-IR photonic devices.

## Teaser

Long-range surface polaritons in 2D material-based structures enable long, high-speed mid-IR photonic transport.

## INTRODUCTION

The field of mid-infrared (mid-IR) photonics offers a multitude of application domains of technological relevance, spanning from gas detection, biomedical imaging, free-space communication, infrared astronomy, and thermal inspection and analysis (*1*–*7*). However, efficient interactions between free-space mid-IR light and molecules are limited by the considerable size disparity, hindering the development of miniaturized and portable mid-IR devices. Recent advances in the search for polaritonic modes in anisotropic crystals (*8*–*18*), such as graphene plasmons (GPs) (*19*–*27*) and hyperbolic phonon polaritons (HPhPs) (*12*, *28*–*35*) supported by two-dimensional (2D) van der Waals (vdW) materials, have been offering interesting solutions to enhance mid-IR light-matter interactions at the nanoscale.

However, both GPs and HPhPs suffer from limited propagation lengths. For instance, GPs in the mid-IR range can propagate only over a few micrometers at room temperature because of intrinsic plasmonic scattering channels (*36*). HPhPs in 2D vdW materials can hardly propagate longer than 20 μm because of the material absorption loss, and they often carry large momenta with slow group velocities (*37*, *38*). To realize compact and planar mid-IR photonic systems incorporating these elements as building blocks for sensing, processing, and computing, it becomes imperative to develop highly efficient, high-speed, and miniaturized platforms for polariton transport across long distances. As possible candidates, ghost hyperbolic surface polaritons have been recently investigated to realize directional, diffraction-less, and long-range propagation (more than 20 μm) along a crystal interface (*35*). However, despite the evanescent decay away from the interface, these polaritons extend up to a few micrometers into the crystal bulk, contributing to the propagation loss. Leaky polaritons, another type of nonuniform surface waves, also support long-range propagation in the mid-IR regime, yet they are strongly hybridized with bulk radiative states (*37*). As a result, both of them require substantially thicker crystals [for instance, 500-μm calcite samples in (*35*)] and suffer from unnecessary material and radiative loss.

In this work, we harness the absorption line around a strong phonon resonance to attain long-range surface polaritons in thin layers, matched in terms of modal profiles to conventional slower HPhPs for facile mode conversion. Realizing such long-propagating surface polaritons over low-dimensional materials at mid-IR frequencies may enable high-speed and highly efficient polariton routing and interconnects (*39*) in ultracompact integrated mid-IR photonic systems. In the following, we demonstrate mid-IR long-range surface polaritons (LRSPs) supported by a heterostructure with a deep-subwavelength thickness (∼1/14.2λ_0_), consisting of a thin hexagonal boron nitride (h-BN) flake, a high-index germanium (Ge) layer, and a gold (Au) film. We exploit the large imaginary part of permittivity around the phonon vibration line of h-BN, whose polariton dispersion is modified and controlled by the high-index substrate and the Au film. The heterostructure is shown to support a substantial LRSP propagation distance, longer than the entire h-BN flake, exceeding 80 μm. This achievement highlights the potential of LRSP based on 2D vdW heterostructures as a promising solution for long-distance on-chip mid-IR polariton transport and seamless integration with other vdW material–based mid-IR photonic devices.

## RESULTS

### Heterostructure design

We consider an interface at *z* = 0 between the free space (*z* > 0) and a nonmagnetic material characterized by a uniaxial relative permittivity tensor $\overset{=}{\varepsilon }$ $=\text{diag}\left(\varepsilon_{t},\varepsilon_{t},\varepsilon_{z}\right)$. The surface polaritons of interest in this work are transverse-magnetic (TM) modes confined to the interface and emerging for negative values of in-plane permittivity, characterized by the magnetic field $H_{0}=\hat{y}H_{y0}e^{ik_{t}x}e^{ik_{0z}z}$ in the free space and $H_{1}=\hat{y}H_{y0}e^{ik_{t}x}e^{ik_{1z}z}$ in the anisotropic medium. *H*_*y*0_ is the field amplitude, and *k_t_* and *k*_0,1*z*_ are the longitudinal and transverse momenta, respectively. The polariton dispersion follows (see Supplementary Text for detailed derivations) (*40*, *41*)

$$\frac{k_{t}}{k_{0}}=\sqrt{\frac{\varepsilon_{t}-1}{\varepsilon_{t}-\varepsilon_{z}^{-1}}}$$ (1)

where *k*_0_ = 2π/λ is the wave number in vacuum, and λ is the free-space wavelength. When Im(ε*_t_*)/[−Re(ε*_t_*)] ≫ 1, Zenneck-type surface polaritons can be supported at the interface, with the transverse momentum closer to the light line *k_t_* ≈ *k*_0_. These polaritons feature a small decay rate [Im(*k_t_*) ≪ Re(*k_t_*)], thus a long propagation length. In the following, we demonstrate mid-IR LRSPs harnessing the strong transverse optical (TO) phonon resonance in the upper Reststrahlen band (ω = 1370 to 1610 cm^−1^) of thin h-BN flakes. In particular, such LRSPs emerge when the involved material exhibits a strong absorption resonance, which results in in-plane permittivity with a large imaginary part and a negative real part. In the mid-IR range, h-BN is an attractive material for LRSPs, as it features a sharp absorption line, stemming from TO phonon vibration in its Reststrahlen bands (*29*, *42*). In addition, as a vdW material, the h-BN crystal can be easily integrated into 2D heterostructures with other 2D materials for ultracompact devices.

**Fig. 1.**
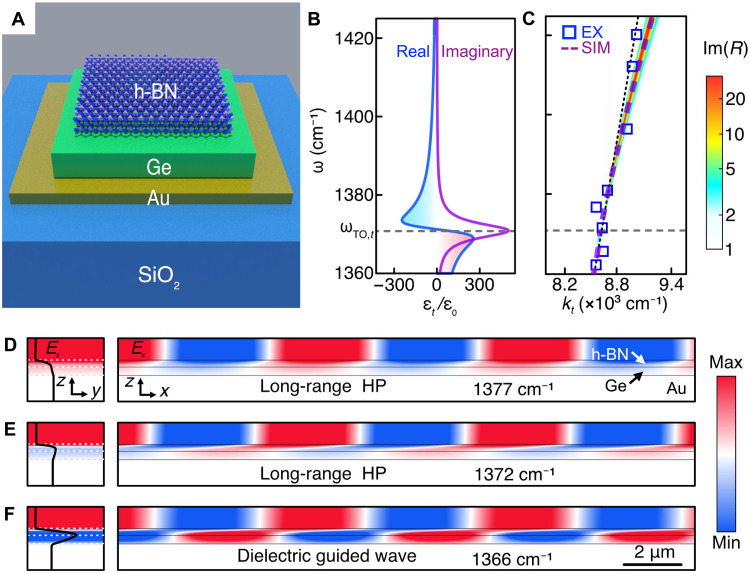
LRSPs on an h-BN-substrate heterostructure. (**A**) Schematic of the sample configuration. (**B**) In-plane relative permittivity (ε*_t_*/ε*_0_*) of h-BN in the upper Reststrahlen band. The blue curve represents the real part, and the purple curve depicts the imaginary part. The blue (purple) shading emphasizes the regime of positive (negative) permittivity above (below) the TO phonon frequency ω_TO,*t*_ = 1370 cm^−1^. (**C**) Dispersion of LRSPs supported by the heterostructure. The experimental results from near-field measurements are labeled by blue square symbols (EX), overlapped with the dispersion diagram from COMSOL simulations (purple dashed curve; SIM) and the color map of the calculated imaginary part of the reflection coefficient Im(*R*), as a function of frequency ω and in-plane wave number *k_t_*. The dotted black line denotes the light cone. (**D** to **F**) Modal fields at three different frequencies around the phonon resonance. The black curves in the left images of (D) to (F) illustrate the variation of the longitudinal electric field *E_x_* along the *z* direction.

As a downside to using h-BN crystals alone, the thickness of h-BN should substantially exceed the skin depth [∼1/Im(*k*_1*z*_), about 100 nm in the upper Reststrahlen band (*43*)] to support LRSPs without substantial radiation leakage. In practice, at least half a micrometer is required for a pure h-BN structure. To address this issue, we introduce a metal reflector beneath the h-BN flake to reduce the required thickness of h-BN to support LRSPs (see Supplementary Text for the detailed discussion). Here, we consider a 2D heterostructure formed by an h-BN-dielectric-reflector stack, as shown in Fig. 1A. Our optimized design can be modeled as a stratified structure consisting of h-BN flake with a thickness of 177 nm on top of a 293-nm-thick Ge layer, supported by a Au film (∼100 nm). The atomic force microscope (AFM) and optical images of the h-BN layer are shown in fig. S12A.

The large phonon-induced resonance of h-BN sustains the LRSP propagation. As shown in Fig. 1B, the real part of the in-plane permittivity Re(ε*_t_*) is smaller than zero at ω = 1372 cm^−1^, while the imaginary part [Im(ε*_t_*)] is as large as 310. Equation 1 provides *k_t_* ≈ (0.999 + 0.0017i)*k*_0_ and then *k*_0*z*_ ≈ (0.059 + 0.029i)*k*_0_ for the wave supported by a semi-infinite h-BN slab at this frequency, corresponding to an LRSP with a wavelength slightly longer than the one of the free space. Phenomenologically, such an LRSP is expected to partially penetrate the thin slab if we suspend the assumed infinitely thick h-BN, because the thickness of the slab is comparable to the skin depth as aforementioned. Hence, the LRSP decays gradually along its propagation because of the leakage downward. The presence of the high-index dielectric (Ge) layer and the Au mirror acts as a reflector preventing such slight leakage and confining the field below the interface within the heterostructure. This effect allows polaritonic phenomena to arise and, at the same time, preserves the long propagation distance of LRSPs. The dispersion of LRSPs in this heterostructure can be analytically determined by calculating the reflection spectra by the transfer matrix method (see Supplementary Text for details). The imaginary part of the reflection coefficient Im(*R*) as a function of frequency ω and transverse momentum *k_t_* is shown as a background in Fig. 1C. The loci of its maxima indicate the modal dispersion, which agrees well with the dispersion diagram (dashed purple curve) from modal analysis in COMSOL Multiphysics. All LRSPs remain quite close to the light cone (black dotted line), confirming their fast group velocity. At the same time, this also corresponds to a poor lateral confinement of these LRSPs, which is an inherent trade-off required to minimize propagation loss, illustrating a fundamental balance between confinement and propagation length in polaritonic systems. Even though weak confinement is not ideal for high-density on-chip integration, the long propagation distance, which far exceeds those of strongly confined HPhPs or GPs in 2D vdW materials, is well suited for interconnects. As such, we believe that the proposed polaritons can enable fast, long-range surface-wave transport in the mid-IR for applications as interconnects in micrometer-scale mid-IR photonic chips and other planar devices based on 2D vdW materials.

Only the modes above the TO phonon frequency ω_TO,*t*_ = 1370 cm^−1^ [horizontal gray dashed line in Fig. 1 (C and D)] can be referred to as LRSPs, corresponding to the region with a negative real part of in-plane permittivity for h-BN, as shaded in purple in Fig. 1B. We compare the modal electric field *E_x_* around the phonon resonance in Fig. 1 (D to F). It can be seen that the fields at ω = 1377 cm^−1^ (Fig. 1D) and ω = 1372 cm^−1^ (Fig. 1E) notably peak at the interface between h-BN and air, exhibiting a polaritonic profile. The LRSPs show oblique wavefronts inside the h-BN and the dielectric layer resulting from the complex-valued wave vector *k*_1*z*_ and consistent with ghost polaritons in (*35*), yet with much longer propagation distances. Without the h-BN layer, the fundamental mode of the Au-backed thin dielectric layer would be almost a plane wave with a wave vector parallel to the interface, while much less field can penetrate and interact with the gold as *k*_0*z*_ ≈ 0. In this scenario, only incident waves almost perfectly parallel to the surface can excite such a quasitransverse electromagnetic mode. By contrast, the presence of strong phonon vibrations of h-BN at ω = 1372 cm^−1^ tilts the polariton wavefronts in the h-BN layer, which makes LRSPs nonuniform TM waves weakly confined at the surface and slowly decaying along the transverse direction as *k*_0*z*_ > 0 and, thus, accessible from far-field excitations. Moreover, the field near resonance, as shown in Fig. 1E, is more confined within the h-BN and penetrates less to the dielectric, leading to lower absorption and, thus, longer propagation distance (∼925 μm) compared to the one at ω = 1377 cm^−1^ depicted in Fig. 1D (∼388 μm). Figure 1F shows the modal field at 1366 cm^−1^ (below the phonon resonance), at which the field peaks at the interface between h-BN and the dielectric and occupies the entire region underneath the air. In contrast, the h-BN now behaves as a dielectric with a large positive Re(ε*_t_*) ≈ 234, making the heterostructure analogous to a Au-backed high-index dielectric waveguide, which supports a TM-polarized weakly guided mode (*44*) rather than a polariton mode. The propagation distance becomes considerably shorter, given that the dielectric loss now becomes dominant. It should be noticed that the weakly confined nature of LRSPs makes them susceptible to sudden out-of-plane curvature changes on surfaces. However, the proposed applications mainly involve the integration of other 2D vdW material–based mid-IR photonic devices and flat nanostructures, so relatively large curvatures can be prevented. In addition, it is possible to design, e.g., an interferometer setup based on weakly localized surface waves, as demonstrated in (*39*).

In some ways, the plasmonic counterpart of the mid-IR LRSPs studied here is the long-range surface plasmon polariton (LRSPP) supported by a very thin metal film embedded in symmetric or asymmetric dielectric/metal/dielectric structures (*45*, *46*). LRSPPs have been extensively studied over the past several decades. However, their physical origin is different from the mid-IR LRSPs reported in this work. In particular, LRSPPs arise from the coupling of single-interface surface plasmon polaritons supported at the two metal-dielectric interfaces of a sufficiently thin metal film. This coupling gives rise to a long-range mode with substantially reduced overlap with the lossy metal, thereby substantially decreasing propagation loss. By contrast, the LRSPs studied here are Zenneck-type surface polaritonic modes supported by thin flakes of polar vdW crystals in the mid-IR range, and their propagation is strongly influenced by the intrinsic TO phonon resonances.

### Near-field observation of LRSPs

**Fig. 2.**
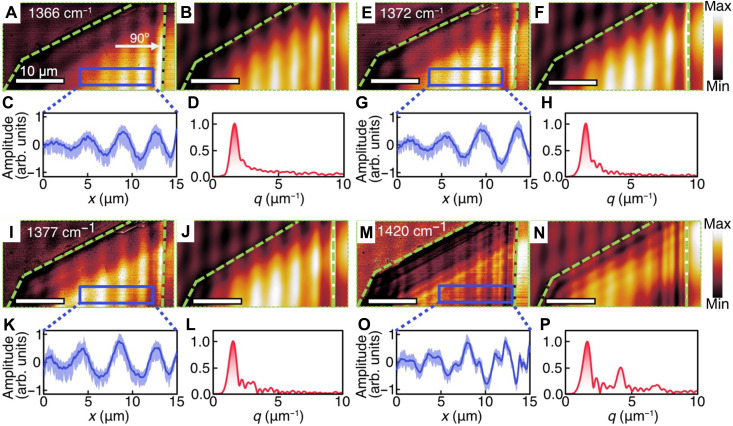
Real-space near-field images of LRSPs and fringe spectra. (**A**) Real-space near-field image measured in the experiment at 1366 cm^−1^. The azimuth angle of the incident beam to the right edge of the h-BN flake is ∼90°, as labeled by the white arrow. (**B**) False-color plot of the magnitude of the electric field obtained from FDTD simulations at the same frequency. (**C**) Normalized fringe plot after removing the background trends in the real space. The shading indicates the range of the normalized *S*_3_ signals along the horizontal direction (*x* axis), taken from each horizontal line that cuts in the range of the blue box in (A), and the blue curve shows the average fringe amplitude. (**D**) Spectrum of the fringe field profile in the reciprocal space (*q*) as the amplitude of the Fourier transform of the average amplitude in (C). (**E** to **P**) Same for the experiments and simulations at 1372 cm^−1^ [(E) to (H)], 1377 cm^−1^ [(I) to (L)], and 1420 cm^−1^ [(M) (P)]. Scale bars are 10 μm.

Real-space near-field images of LRSPs obtained from a scattering-type scanning near-field optical microscope (s-SNOM) are shown in Fig. 2. The plots of the third harmonic of the tapping frequency (*S*_3_) clearly show the fringes for 1366 cm^−1^ (7.32 μm) to 1420 cm^−1^ (7.04 μm), as observed in Fig. 2 (A, E, I, and M). They match well with the magnitudes of the electric field from finite-difference-time-domain (FDTD) simulations (see Materials and Methods). After removing the background trends of *S*_3_ signals (*47*), the normalized interference fringes are shown in Fig. 2 (C, G, K, and O). The solid curves denote the average and normalized fringe amplitude in the blue boxes, after removing the background trends, as a function of the horizontal direction, and the blue shadows shade the range of the normalized amplitudes taken along many horizontal cut lines in the blue boxes.

Because the wavelength of LRSPs is close to the free-space light cone, the fringes observed are derived from the interference between the edge-launched LRSPs and the oblique incident light. It should be mentioned that this interference and the demodulation of the detector output enable the observation of LRSPs in s-SNOM measurements and their separation from the incident free-space plane wave despite their very similar field dispersion in air, as illustrated in Fig. 1 (D and E) (detailed discussion is in Materials and Methods and Supplementary Text). Hence, rather than directly taking the peak-to-peak distance as λ/2 under the assumption of the interference of the tip-launched polariton and the edge-launched one, we take the Fourier transform of the fringes, as shown in Fig. 2 (D, H, L, and P). The peaks in the reciprocal space (red curves) indicate the prominent momentum components carried by the fringes. We then obtain the momenta *k_t_*’s of LRSPs by calibrating the interference between the free-space light with an incident angle around 60° and the LRSPs launched from the h-BN flake edge, as schematically illustrated in fig. S9 and explained in Supplementary Text. These retrieved momenta are denoted by blue squares in Fig. 1C.

**Fig. 3.**
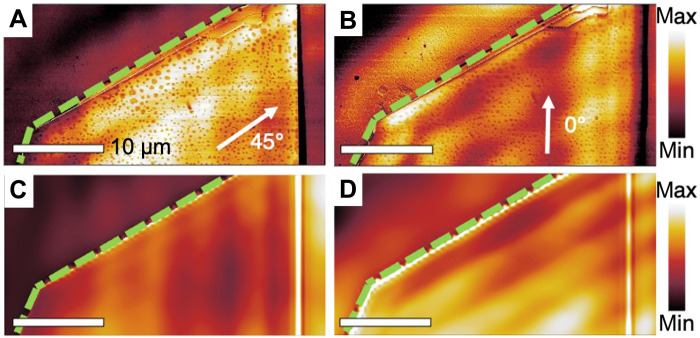
Real-space near-field imaging of LRSPs at 1372 cm^−1^. (**A** to **D**) The images in (A) and (B) are experimental results, and those in (C) and (D) are obtained from FDTD simulations. The azimuth angles of the incident beam with respect to the right edge of the h-BN flake are 45° [(A) and (C)] and 90° [(B) and (D)]. Scale bars are 10 μm.

In Fig. 2P, we observe two types of fringes, with both a large peak-to-peak distance and an apparently shorter distance. This observation is consistent with the additional peak that appears around 4.17 μm^−1^ in the spectra shown in Fig. 2O, indicating the excitation of not only the LRSPs but also of higher-order HPhPs (*38*, *48*) at 1420 cm^−1^. This higher-order mode is clearly observed in the momentum spectra at frequencies above 1380 cm^−1^, as discussed in Supplementary Text and shown in fig. S10, and in the extended dispersion plot in fig. S11. As the frequency approaches ω_TO,*t*_, its damping rate increases, and thus, no higher-order HPhPs can be observed near ω_TO,*t*_ (*29*). The experimental results attained from a bare h-BN flake, illustrated in fig. S13, confirm that there is no near-field fringe observed at and near 1372 cm^−1^, which also rules out the possibility that the observed fringes in Fig. 2 are derived from cylindrical waves launched by the h-BN flake edge (*49*). The measured in-plane momenta of LRSPs at different frequencies are denoted by the blue squares in Fig. 1C, confirming that the in-plane momenta (*k_t_*) are close to the light line. As shown in the left insets of Fig. 1 (D and E), the *E_x_* component depicts that LRSPs reach their maximum value at the interface between the strongly lossy medium (h-BN) and air and have negligible penetration to the lossy media, which promises extremely small decay along its propagation. As shown in Fig. 3 (A and B), when the azimuthal direction of the incident light is slanted by 45° or 90° with respect to the straight edge of h-BN, there are no well-defined fringes observed along the horizontal direction, which is further verified by the FDTD simulations, as shown in Fig. 3 (C and D).

### Heterostructure optimization

**Fig. 4.**
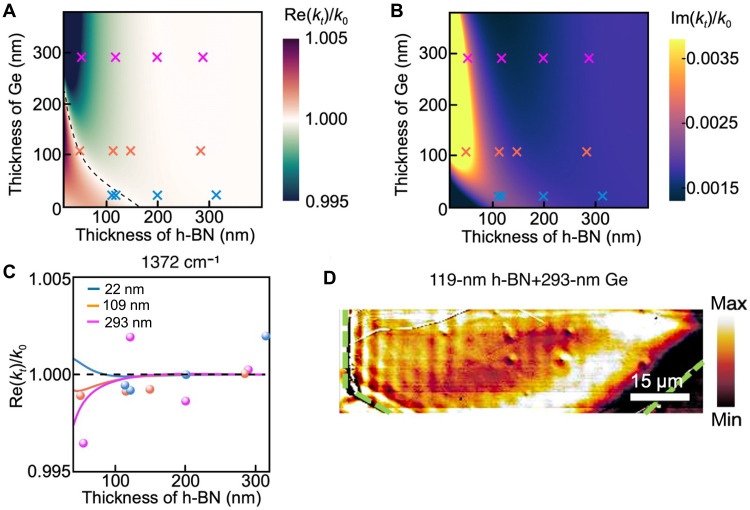
LRSPs at 1372 cm^−1^ on h-BN + Ge + Au samples. (**A**) Dependence of Re(*k_t_*)/*k*_0_ on the thickness of the h-BN flake and Ge layer. (**B**) Dependence of Im(*k_t_*)/*k*_0_, inversely proportional to the propagation length of LRSPs, on the thickness of the h-BN flake and Ge layer. (**C**) Dependence of Re(*k_t_*)/*k*_0_ on the thickness of the h-BN flake when the thicknesses of Ge are 22 nm (blue), 109 nm (orange), and 293 nm (pink). The dashed back line labels the light line. (**D**) Real-space near-field image of 119-nm h-BN + 293-nm Ge sample. The scale bar is 15 μm.

Having demonstrated the emergence of LRSPs in the proposed 2D vdW material–based heterostructures, we aim to explore how long the propagation length can be extended for mid-IR photonic applications. First, we analytically explore the propagation of LRSPs characterized by the in-plane momentum *k_t_*, whose real part is related to the phase velocity, while the imaginary part captures the decay along the wave propagation. By optimizing the thickness of the h-BN flake and of the Ge layer, we explore low-loss and fast LRSPs in the proposed heterostructure. The calculated dispersion diagrams at 1372 cm^−1^ are shown in fig. S4, verifying that LRSPs are possible around this frequency with Re(*k_t_*) approaching *k*_0_. The variations of Re(*k_t_*)/*k*_0_ and Im(*k_t_*)/*k*_0_ for different thicknesses of Ge and h-BN are shown in Fig. 4 (A and B, respectively). The black dashed curve in Fig. 4A indicates the contour of the light line Re(*k_t_*) = *k*_0_. Experimentally, we studied samples with three different Ge thicknesses: 22, 109, and 293 nm, each stacked with h-BN flakes of different thicknesses, as indicated by the crosses in the figure. The values of Re(*k_t_*)/*k*_0_, obtained from the measured fringe distances in the near-field amplitude images, are shown in Fig. 4A, and they follow the same trend as the analytical results (solid curves in Fig. 4C). In particular, when the h-BN thickness decreases below 100 nm, the values of Re(*k_t_*)/*k*_0_ deviate more rapidly from the light line [Re(*k_t_*)/*k*_0_ = 1], resulting in higher loss and shorter propagation lengths. In addition, this deviation is more pronounced for the thicker Ge layer (293 nm, pink curve) than for the thinner one (109 nm, the orange curve), as revealed in Fig. 4C. The quantitative deviations between the experimental results and the theoretical curves may arise from imperfections in sample preparation and experimental measurements. Although some of them are fast waves (Ge: 109 nm; Ge: 293 nm), these LRSPs suffer from larger decay, entering the bright region in Fig. 4B with larger Im(*k_t_*), which may be due to leakage. Therefore, an h-BN flake with a thickness larger than 100 nm is required to support LRSPs. To investigate the effect of the Ge layer thickness, we prepared samples with ∼100-nm h-BN and different Ge thicknesses. The analytical results in Fig. 4B illustrate that Im(*k_t_*) in the fast-wave region remains minimal if the thickness of h-BN flakes is around 100 to 200 nm and the one of Ge is larger than ∼200 nm. Accordingly, we chose 119-nm h-BN on top of 293-nm Ge and 100-nm Au (total transverse footprint ∼1/14.2λ_0_) to demonstrate long-range 2D transport of mid-IR polaritons. The near-field image in Fig. 4D reveals that mid-IR LRSPs can propagate across an entire h-BN flake with a propagation length longer than 80 μm. The right edge of the h-BN flake in Fig. 4D is intentionally chosen to have a small azimuthal angle to the incident light to avoid the generation of LRSPs from the right edge. The AFM and optical images of this h-BN flake are shown in fig. S12B. The measured propagation length is limited by the size of the h-BN flake, which implies that the observed LRSPs can propagate a notably longer distance than HPhPs and GPs. This feature also indicates that the potential applications of LRSPs may be restricted by the realistic size of available h-BN flakes, but we note that previously unknown techniques are emerging for producing large-area h-BN flakes, such as those reported in (*50*, *51*). It should be mentioned that the maximum scanning size of our s-SNOM is 100 μm by 100 μm, which also limits the propagation distance demonstrated. To compare with other thickness choices and justify our analytical optimization, near-field images are presented in fig. S14 for other samples, showing that both the near-field fringes on the 120-nm h-BN + 22-nm Ge and 114-nm h-BN + 109-nm Ge samples decay more swiftly, with a propagation length of less than 15 μm. The near-field image of the 184-nm h-BN + 293-nm Ge sample in fig. S15 also demonstrates that LRSPs can travel across a large h-BN flake with no substantial decay, consistent with the predictions in Fig. 4B.

It should be noted that a figure of merit, which may be defined as the quality factor Q=Re(k)/Im(k) (*38*), provides a more comprehensive assessment of polaritonic performance. In our experiments, Re(k)≈0.8634 μm^−1^ (Fig. 4C), while $\text{Im}\left(k\right)=1/\left(2L_{p}\right)$, where $L_{p}$ denotes the propagation length. Accordingly, the figure of merit can be expressed as $Q=\text{Re}\left(k_{t}\right)\cdot 2L_{p}$. However, we are unable to accurately extract an experimental value of $L_{p}$, because the LRSPs extend to the opposite edge of the h-BN flake, as shown in Fig. 4D. As a result, a direct determination of the intrinsic propagation length is limited by the finite size of the available h-BN flakes. Alternatively, we estimate a lower bound for the quality factor using the longest LRSP propagation distance observed (∼80 μm) in the 119-nm h-BN + 293-nm Ge sample. This yields an estimated lower bound of the quality factor $Q_{\text{min}}\approx 138$ at 1372 cm^−1^, which exceeds the reported values Q<50 for HPhPs in h-BN (*38*) and Q<20 for GPs (*10*). In theoretical calculations, the *Q* factor for LRSPs in the 119-nm h-BN + 293-nm Ge sample is $0.9998/\left(8.174\times 10^{-3}\right)\approx 1223$ [data point from Fig. 4 (A and B): $\frac{k_{t}}{k_{0}}\approx 0.9998+8.174\times 10^{-3}i$] at 1372 cm^−1^.

## DISCUSSION

In this work, we demonstrated fast LRSPs on 2D h-BN-dielectric-metal heterostructures in the mid-IR range, experimentally achieving propagation distances longer than 80 μm across a whole h-BN thin flake and theoretically predicting a propagation distance of ∼925 μm. The utilization of LRSPs relies on the large imaginary part and negative real part of the h-BN thin flake’s permittivity derived from TO resonance to support fast wave propagation along the interface. By leveraging LRSPs, we can overcome the limitations imposed by the propagation length of confined polaritons, ideally suited to bridge polariton transport along long distances on chips. The integration of 2D LRSP waveguides into mid-IR photonic devices holds great promise for enabling efficient and high-speed on-chip polariton interconnects, providing a promising paradigm for the development of advanced mid-IR integrated photonic systems and miniaturized mid-IR photonic devices.

## MATERIALS AND METHODS

### Numerical simulations

The eigenmode simulations of the LRSPs were performed in COMSOL Multiphysics. The numerical port boundary conditions are applied to obtain the complex eigenwave number (*k_t_*) corresponding to a given real frequency ω. Only in-plane (*xz* plane) polarization is calculated in the 2D models in COMSOL, which represents the TM polarization of our interest. The simulations of interfering fringes are performed in Ansys Lumerical FDTD. 3D models of the stratified media are studied in the simulations, impinged by a TM-polarized Gaussian pulse whose wavelength spectrum spans from 6 to 8 μm. The magnitudes of the electric field component normal to the interface are recorded for the plots.

### Sample preparation

To prepare the h-BN + Ge + Au heterostructures, a Au film with an ∼100-nm thickness is deposited on a 300-nm silicon dioxide (SiO_2_) layer on a silicon substrate, and then an amorphous Ge layer is deposited on the Au film via an e-beam evaporator. h-BN flakes were transferred to the top surface of the Ge or SiO_2_ layer via a conventional dry transfer method.

### s-SNOM measurements

An s-SNOM (Neaspec GmbH) in the reflection mode was used to obtain the real-space near-field amplitude images. A tunable continuous wave quantum cascade laser (MIRcat-QT, DRS Daylight Solutions) was used as the excitation source. A commercial AFM tip with a PtIr5 coating (NanoWorld) is used as the probe. The images were obtained via a pseudoheterodyne interferometric detection module with an AFM tapping amplitude of ∼70 nm and at the third harmonic of the tapping frequency (*S*_3_), which is ∼285 kHz.

### Origin of near-field fringes of LRSPs

To isolate near-field signals from free-space contributions, a lock-in amplifier demodulates the detector output at higher harmonics of the AFM tip tapping frequency, leveraging the strong nonlinearity of near-field interactions. The third harmonic signal (*S*_3_) originates from either evanescent surface modes or tip-sample near-field coupling. The latter is described by models such as the point-dipole model, where a polarizable sphere under an incident field interacts electrostatically with the sample surface, as schematically illustrated in fig. S5A. The effective polarizability ($\alpha_{\text{eff}}$) is given by the equation below (*52*)

$$\alpha_{\text{eff}}\equiv \frac{P_{z}}{E_{\text{inc}}}=\frac{\alpha }{1-\alpha \beta /\left[16\pi {\left(a+d\right)}^{3}\right]}$$

where $\alpha \equiv 4\pi \varepsilon_{0}a^{3}$ is the bare polarizability of an ideal metallic sphere of radius a in vacuum, *d* is the tip-sample distance along the *z* direction, and $\beta \equiv \frac{\varepsilon \left(\omega \right)-1}{\varepsilon \left(\omega \right)+1}$ is the quasistatic limit of the Fresnel coefficient, describing the *p*-polarized light reflected from the sample surface with permittivity ε(ω). This relationship allows s-SNOM to probe surface-bound modes or map spatial variations in dielectric properties.

From the above equation, the observation of near-field fringes from LRSPs, despite their nonexponential decay in air, can be explained by the dependence of near-field signals on the local field $E_{\text{inc}}$. Specifically, LRSPs are efficiently launched by the edge of the h-BN flake with a field strength comparable to the incident wave, which leads to interference between the propagating LRSPs and the incident light, producing a sinusoidal variation in $E_{\text{inc}}$, as schematically illustrated in fig. S7A. Consequently, s-SNOM detects near-field fringe patterns of LRSPs. More discussion is in Supplementary Text.

## Supplementary Materials

### This PDF file includes:

Supplementary Text Figs. S1 to S15 References
