# Supplementary material for: Mid-infrared long-range surface polaritons in two-dimensional van der Waals materials
Source: Sci Adv. 2026 Aug 5;12(32):eaed2198. doi: 10.1126/sciadv.aed2198 (PMC13440431; doi:10.1126/sciadv.aed2198)
Supplement: Supplementary file 1 [file sciadv.aed2198_sm.pdf]

Science Advances
AAAS

Supplementary Materials for

Mid-infrared long-range surface polaritons in two-dimensional van der Waals materials

Mingsong Wang *et al.*

Corresponding author: Andrea Alù, aalu@gc.cuny.edu

Sci. Adv. **12**, eaed2198 (2026)
DOI: 10.1126/sciadv.aed2198

This PDF file includes:

Supplementary Text
Figs. S1 to S15
References

Supplementary Text

I. Long-Range Surface Polaritons above a semi-infinite lossy medium

As shown in Fig.1A, consider that $z = 0$ is the interface between free space ($z > 0$) and a generic nonmagnetic material characterized by a uniaxial relative permittivity tensor $\bar{\bar{\varepsilon}} = \mathrm{diag}(\varepsilon_t, \varepsilon_t, \varepsilon_z)$. In this section, we will show that there exists a transverse-magnetic (TM) polarizd wave solution with momentum close to the light line — long-range surface polaritons (LRSPs), which have extremely low loss along its propagation. With the imaginary part of the in-plane permittivity ε_t becomes sufficiently large, it features a longitudinal phase velocity faster than the speed of light, strikingly different from the propagation of conventional hyperbolic phonon polaritons (HPhPs) with large momenta and slow group velocities.

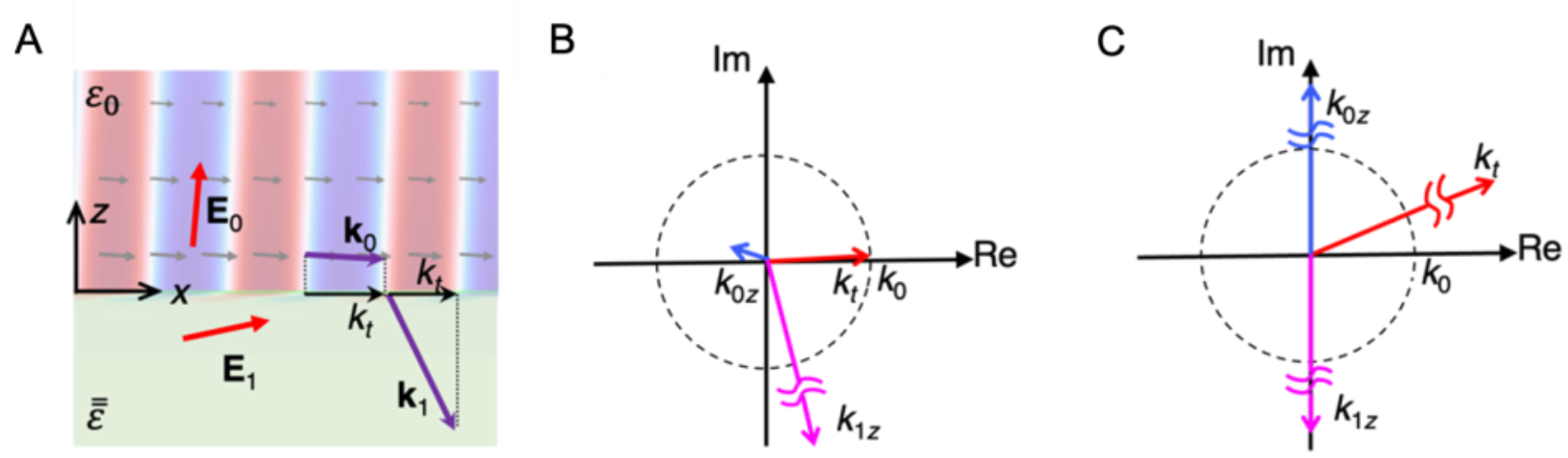

Figure S1 | Long-range hyperbolic polairtons above a semi-infinite lossy medium. (**A**) A long-range surface hyperbolic polariton wave propagating along the interface between free space and a semi-infinite anisotropic and lossy medium. Panels (**B**) and (**C**) show the typical complex wavevectors involved in the propagation of (**B**) LRSPs and (**C**) conventional HPhPs with large momenta. The curly breakpoints indicate the actual length for the quantity exceeds the plot region.

In the free space region ($z > 0$), we assume a magnetic field $\mathbf{H_0} = \hat{y} H_{y0} e^{ik_t x} e^{ik_{0z} z}$, where k_t, k_{0z} are the tangential and normal wavevectors taking complex values in general. The electric field shown in Fig. S1A is then $\mathbf{E}_0 = (k_{0z}\hat{x} - k_t\hat{z}) H_{y0}/(ck\varepsilon_0) e^{ik_t x} e^{ik_{0z} z}$, where c, k, ε_0 are the speed of light, wavenumber, and permittivity in vacuum, respectively. Meanwhile, the dispersion relation of free space reads,

$$k_t^2 + k_{0z}^2 = k_0^2. \quad \text{(S1)}$$

In the lower half space ($z < 0$), the electric and magnetic fields should read $\mathbf{E_1} = (\hat{x} k_{1z}/\varepsilon_t - \hat{z} k_t/\varepsilon_z) H_{y0}/(ck\varepsilon_0) e^{ik_t x} e^{ik_{1z} z}$,and $\mathbf{H_1} = \hat{y} H_{y0} e^{ik_t x} e^{ik_{1z} z}$, which promises the continuity of tangential magnetic field. The continuity of tangential electric field further imposes

$$k_{1z} = \varepsilon_t k_{0z}. \quad \text{(S2)}$$

The TM-polarized wave that we are looking for corresponds to the extraordinary wave in the uniaxial anisotropic medium, whose dispersion relation can be derived from the eigenvalue problem of the wave equation $\nabla \times \nabla \times \mathbf{E}_1 + k_0^2 \bar{\bar{\varepsilon}} \mathbf{E}_1 = 0$, rendering

$$\frac{k_{1z}^2}{\varepsilon_t} + \frac{k_t^2}{\varepsilon_z} = k_0^2. \quad \text{(S3)}$$

Combining Eqs. S1-S3, one can solve for all the three wavevector components k_t, k_{0z}, and k_{1z}, which sufficiently describe the propagation of a wave. In particular, the in-plane wavenumber

$$k_t = k_0 \left(1 + \frac{1-\varepsilon_z^{-1}}{\varepsilon_t - 1}\right)^{-\frac{1}{2}}, \tag{S4}$$

which is essentially Eq. 2 in the main text. The other two wavevector components can further be obtained by $k_{0z} = \sqrt{k_0^2 - k_t^2}$ and $k_{1z} = \varepsilon_t \sqrt{k_0^2 - k_t^2}$.

Now we consider the scenario when the out-of-plane relative permittivity ε_z remains far from the resonance, while the in-plane permittivity ε_t exhibits significant larger imaginary part than its real part, i.e., $\mathrm{Im}(\varepsilon_t) \gg \mathrm{Re}(\varepsilon_t)$, $\mathrm{Im}(\varepsilon_t) \gg |1 - \varepsilon_z^{-1}| > 1$. The binomial expansion of Eq. S3 then estimates

$$k_t \approx k_0 \left[1 - \frac{1-\varepsilon_z^{-1}}{2(\varepsilon_t - 1)}\right] \approx k_0 \left[1 - \frac{1}{2i\mathrm{Im}(\varepsilon_t)}\right], \tag{S5}$$

which suggests a propagating wave with extremely small decay [$\mathrm{Im}(k_t) \ll k_0$] and fast phase velocity [$\mathrm{Re}(k_t) \approx k_0$]. This particular solution is historically referred to Zenneck wave*(53)*, and we propose the LRSPs in the similar spirit in the stratified structure described in the main text and discussed in the following section. An example schematic of the complex wavevectors is shown in Fig. S1B: $\mathrm{Re}(k_t)$ can be even smaller than k_0, which means the phase velocity can exceed the speed of light; $\mathrm{Im}(k_t)$ is extremely small, indicating a long propagating distance $1/[2\mathrm{Im}(k_t)]$ (defined as the distance where the power of the wave decays to 1/*e* of its initial power.) k_{1z} has a sufficiently large imaginary part, guaranteeing the fast decay into the lossy medium. k_{0z} is small but still indicates decay in the free space region. Overall, such values of the three complex wavevector components correspond to a surface wave that is weakly confined to the interface with extremely small propagation loss and fast phase velocity. The background shading of Fig. S1A depicts a typical field distribution of a fast LRSP with extremely low loss, whose wavefront in the lossy medium is oblique.

II. The role of Au film in the stratified structure.

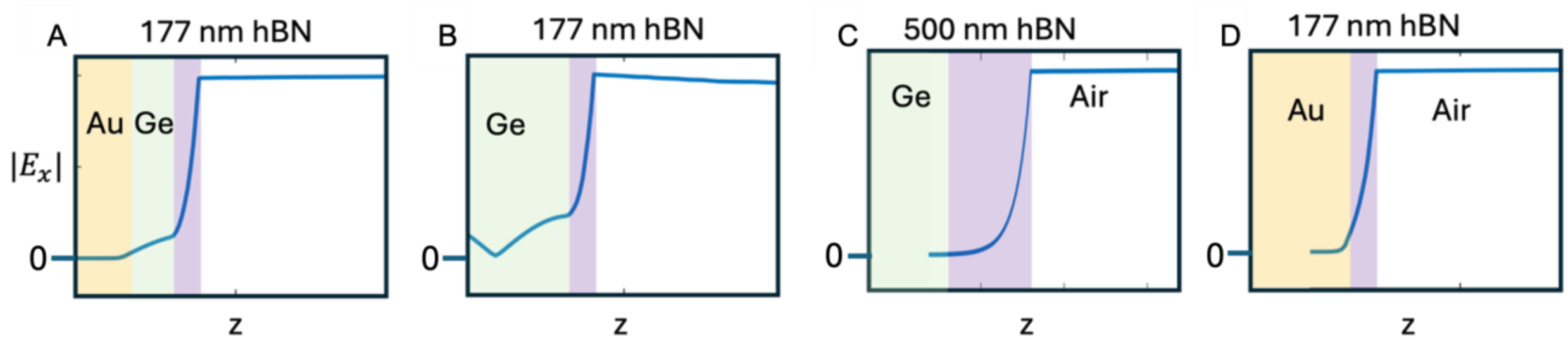

Figure S2 | $|E_x|$ Schematics of stratified structures with different composition. (**A**) 177 nm h-BN-293nm Ge-Au multilayer. (**B**) 177 nm h-BN-Ge bilayer. (**C**) 500 nm h-BN-Ge bilayer. (**D**) 177 nm h-BN-Au bilayer.

As stated in the discussion preceding Fig. S1, the primary role of the metallic reflector is to minimize the required thickness of h-BN to support LRSPs. In order to further illustrate this, the eigenmodes supported by multilayered media comprising different combinations of the h-BN, dielectric substrate (Ge), and the metallic reflector (Au) are juxtaposed in Fig. S2. This figure shows the modal field of LRSPs, in which the field decays exponentially inside the h-BN-Ge-Au multilayer. If the Au layer is removed, as shown in Fig. S2B, the wave leaks into the dielectric layer because the thickness of the h-BN flake barely exceeds the skin depth as discussed in the main text. Thus, this mode cannot propagate through a long distance even though it behaves like a surface wave in the air side. When the thickness of h-BN increases, e.g., the one of 500 nm depicted in Fig. S2C, the field decays sufficiently within the h-BN such that the leakage to the dielectric layer becomes negligible. Obviously, h-BN backed directly with a metallic reflector can support similar modes as well and releases the requirement on the thickness of h-BN, as shown in Fig. S2D. Yet, the dielectric layer was added to engineer the dispersion of LRSPs and to minimize the propagation loss, as discussed in Heterostructure optimization part of the main context.

III. Long-Range Surface Polaritons above a stratified medium

Consider a N-layer stratified medium as shown in Fig. S3A. The thickness of each layer is d_n, and permittivity can generally be anisotropic $\bar{\bar{\varepsilon}} = \mathrm{diag}(\varepsilon_{t,n}, \varepsilon_{t,n}, \varepsilon_{z,n})$ with $n = 1,2,\ldots,N$. A TM-polarized plane wave $H_y = H_0 e^{i(k_t x - k_{0z} z)}$ impinging on Layer 1, as such the out-of-plane wavenumber in each layer $k_{z,n} = \varepsilon_{t,n}\sqrt{k_n^2 - k_t^2}$.

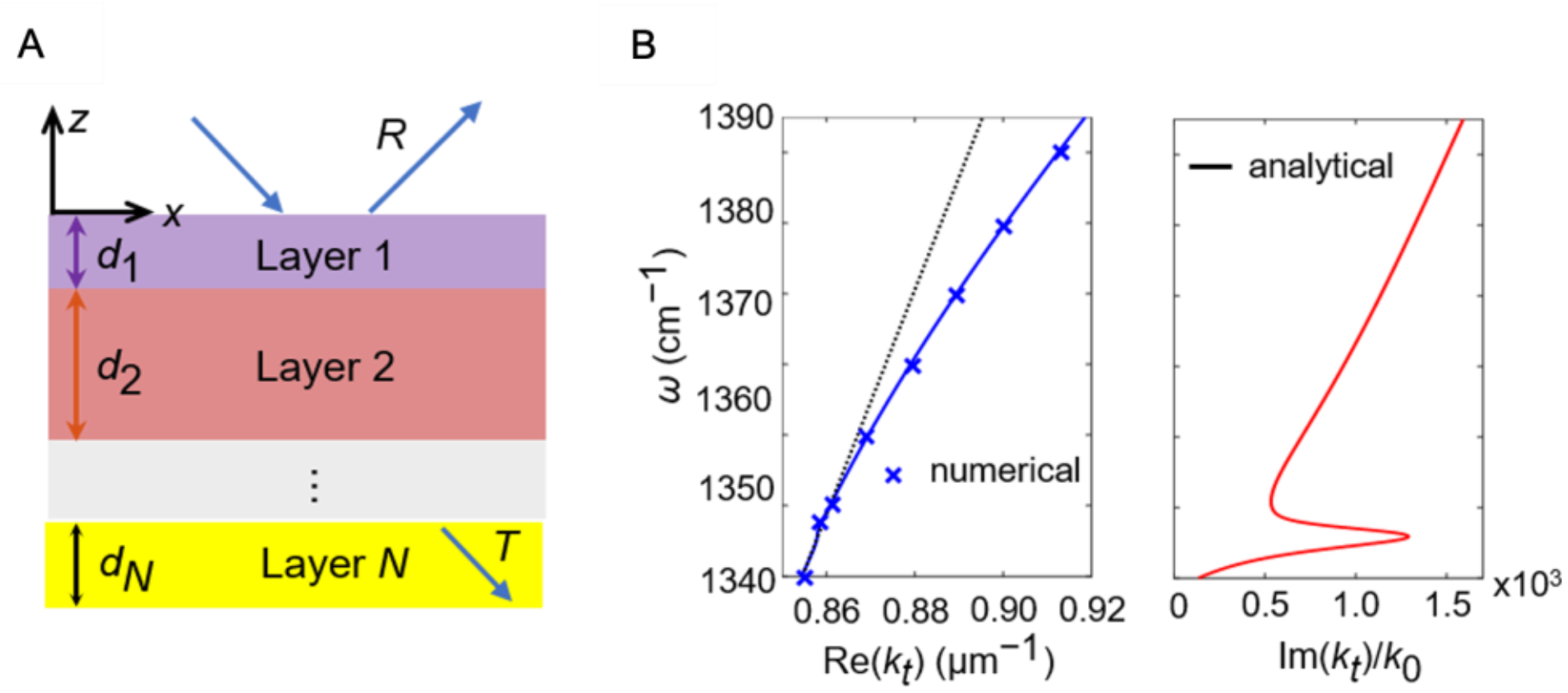

Figure S3 | Layered medium and example of dispersion relations. (**A**) Schematic of a stratified medium of layer N. (**B**) Dispersion relations in the complex k_t domain for the stratified medium discussed in Fig. S1 in the main text.

We then can solve the total reflection R, and transmission T using the transfer matrix method (TMM), which reads,

$$\begin{bmatrix} 1 \\ R \end{bmatrix} = M_{01} \prod_{n=1}^{N-1} (P_n^{-1} M_{n,n+1}) \begin{bmatrix} T \\ 0 \end{bmatrix}, \tag{S6}$$

where

$$M_{n,n+1} = \frac{1}{2\varepsilon_{t,n+1}\mathrm{Re}(k_{z,n})} \begin{bmatrix} \varepsilon_{t,n+1}k_{z,n} + \varepsilon_{t,n}k_{z,n+1} & \varepsilon_{t,n+1}k_{z,n} - \varepsilon_{t,n}k_{z,n+1} \\ \varepsilon_{t,n+1}k_{z,n} - \varepsilon_{t,n}k_{z,n+1} & \varepsilon_{t,n+1}k_{z,n} + \varepsilon_{t,n}k_{z,n+1} \end{bmatrix},$$

$$P_n = \begin{bmatrix} e^{-ik_{z,n}d_n} & 0 \\ 0 & e^{ik_{z,n}d_n} \end{bmatrix}.$$

Continuing k_t to $k_t > k_0$ (below the light cone), one can readily obtain the reflection spectra in the complex-k_t plane.

Eq. S6 indicates that the thickness of each layer in the stratified medium can affect the dispersion of LRSPs. For instance, Fig. S4 shows the dispersion of LRSPs supported by an h-BN-Ge-Au stratified medium, as discussed in the main text. The thickness of h-BN flake is 100 nm, and the thicknesses of Ge are 22 nm (blue), 109 nm (orange), and 293 nm (pink), respectively. For the scenario with 22 nm Ge, we clearly see the strong phonon resonance of h-BN at $\omega = 1370$ cm^{-1} gives rise to LRSPs solutions above the light line (black dashed line). With thicker Ge layer, the LRSPs move closer to the light line. The variation of the decay rates [$\mathrm{Im}(k_t)$], or equivalently, the propagation distances, with different choices of h-BN and Ge layer thicknesses, is discussed in the main text.

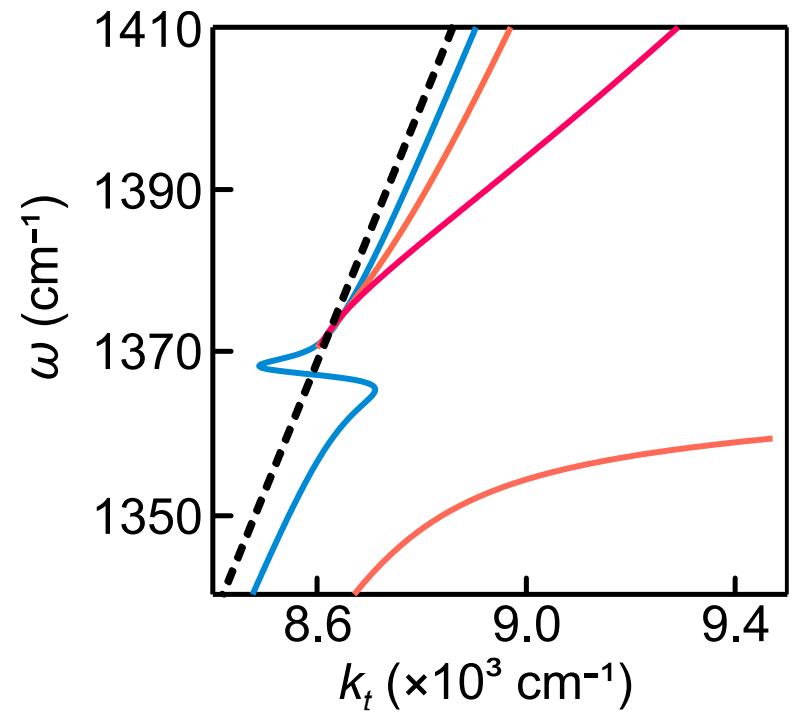

Figure S4 | The dispersion of LRSPs supported by h-BN+Ge samples. The thicknesses of Ge are 22 nm (blue), 109 nm (orange), and 293 nm (pink).

In the quest for the reflection coefficient in Eq. S6, however, the role of layer thickness is simply included in the phase accumulation $e^{\pm i k_{z,n} d_n}$ of the waves. Thus, a thinner dielectric with a high refractive index is expected to play a similar role as a thicker dielectric with a low index, if the electric length $k_{z,n} d_n$ remains the same.

IV. Why near-field fringes of Long-Range Surface Polaritons can be observed

A lock-in amplifier demodulates the detector output at higher harmonics of the AFM tip tapping frequency to isolate near-field signals from free-space contributions via utilizing the strong nonlinearity of near-field interactions. The third harmonic signals (S_3) used in our measurements arise from either evanescent surface modes or tip-sample near-field coupling. The latter is described by theoretical models such as the point-dipole model, where a polarizable sphere under an incident field interacts electrostatically with the sample surface, as schematically shown in Fig. S5A. The effective polarizability (α_{eff}), as given by the equation below*(52)*:

$$\alpha_{\text{eff}} \equiv \frac{P_z}{E_{inc}} = \frac{\alpha}{1-\alpha\beta/[16\pi(a+d)^3]},$$

where $\alpha \equiv 4\pi\varepsilon_0 a^3$ is the bare polarizability of an ideal metallic sphere of radius a in vacuum, d is the tip-sample distance along z direction, and $\beta \equiv \frac{\varepsilon(\omega)-1}{\varepsilon(\omega)+1}$ is the quasistatic limit of the Fresnel coefficient, describing the *p*-polarized light reflected from the sample surface with permittivity $\varepsilon(\omega)$. For instance, stronger S_3 from Si than from SiO_2 at 1372 cm^{-1} indicate higher conductivity in Si, as the near-field amplitude images in Fig. S5A revealed.

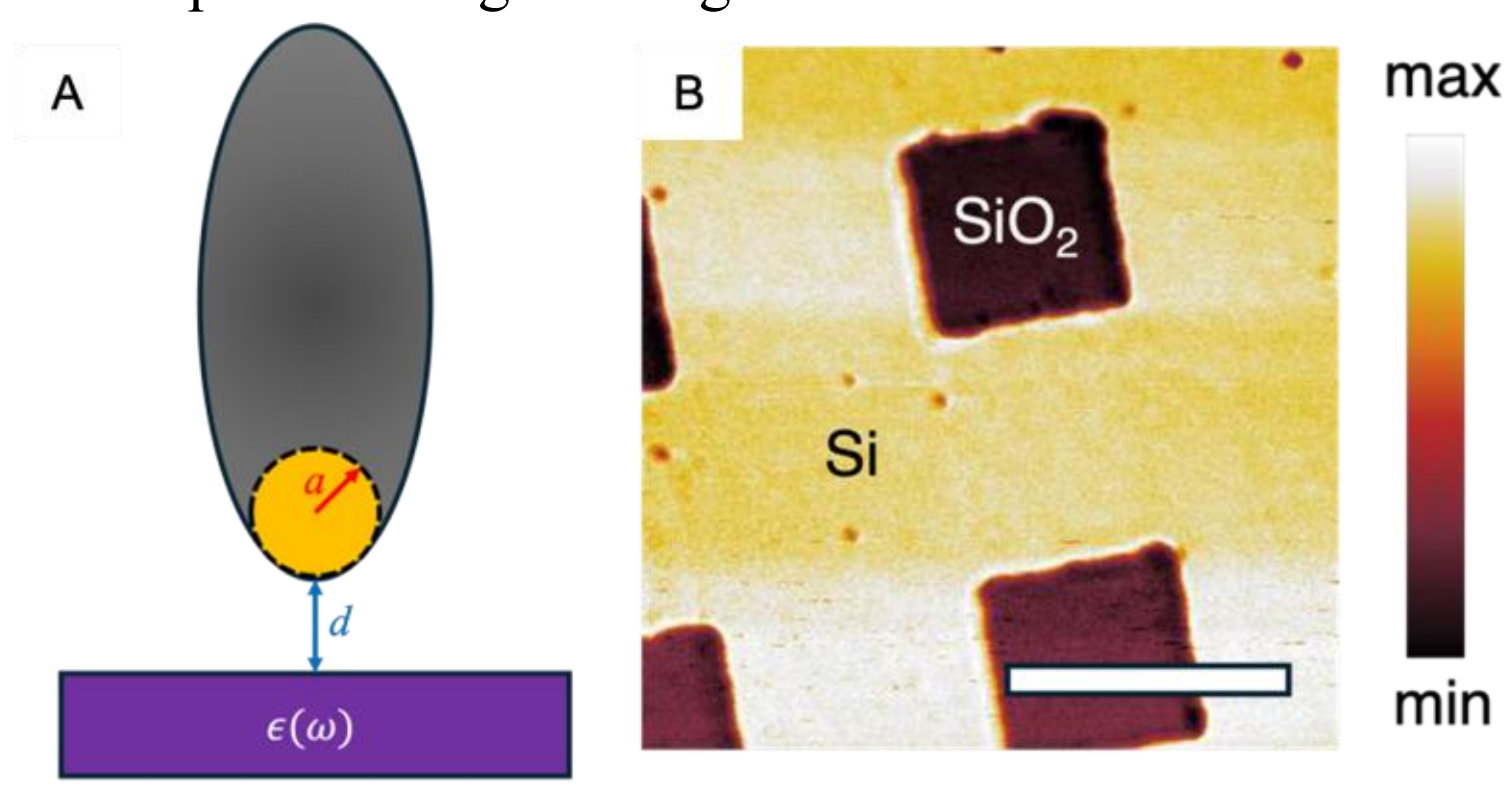

Figure S5 | the tip-sample near-field interaction. a. Schematic of point-dipole model for the tip-sample near-field interaction. b. Real-space near-field images of SiO_2 cubes on a Si wafer at 1372 cm^{-1}. The color bar is 1µm.

To compare the near-field visibility of layered structures with or without h-BN, we implemented in FDTD simulations a gold sphere of 20 nm radius to mimic the AFM tip above different samples and measured its scattering under the same level of plane-wave excitation. As shown in Fig. S6, the scattered signals at the h-BN TO phonon resonance (1370 cm^{-1}) above the proposed multilayers (60 nm above the surface) are significantly stronger than the ones in the absence of h-BN. The pronounced differences in scattering signals arise because, at the TO phonon resonance, h-BN exhibits a significantly larger Im(ε_t) than the Au or Ge layers.

Next, we want to explain why we can observe the near-field fringes of LRSPs, which don't have the exponential decay in the air. From Eq. S7, it is known that the near-field signals depend on the intensity of E_{inc}. In our scenario, the LRSPs can be efficiently launched by the edge of the h-BN flake, whose intensity is comparable to the impinging wave. As a result, E_{inc} is a product of the interference between the free-space incidence and the propagating LRSPs, and thus having a sinusoidal variation in its intensity, as schematically illustrated in Fig. S7A. Therefore, s-SNOM can obtain near-field fringe patterns of LRSPs. Admittedly, as discussed in the main context and

Ref.*(49)*, the edge of h-BN flake can also launch a cylindrical wave into the free space, which could interfere with the incident light and appears in the near-field image, as schematically shown Fig. S7B. But it should be noticed that such scattering-induced cylindrical wave decays at a rate of $1/\sqrt{r}$, where r is the radius from the launcher. Considering the λ_0 is around 6-7μm, the intensity of the cylindrical wave would plummet to much smaller than the incidence within a distance less than a few wavelengths. Therefore, any noticeable interference pattern between the cylindrical wave and the far-field incidence could not be observed via s-SNOM. It should be noted that different from the exponential decay along the out-of-plane tip-sample distance, which is essential for the s-SNOM system to perform demodulation and suppress the far-field background, the decay ($1/\sqrt{r}$) of the cylindrical wave along the in-plane wave propagation direction is different.

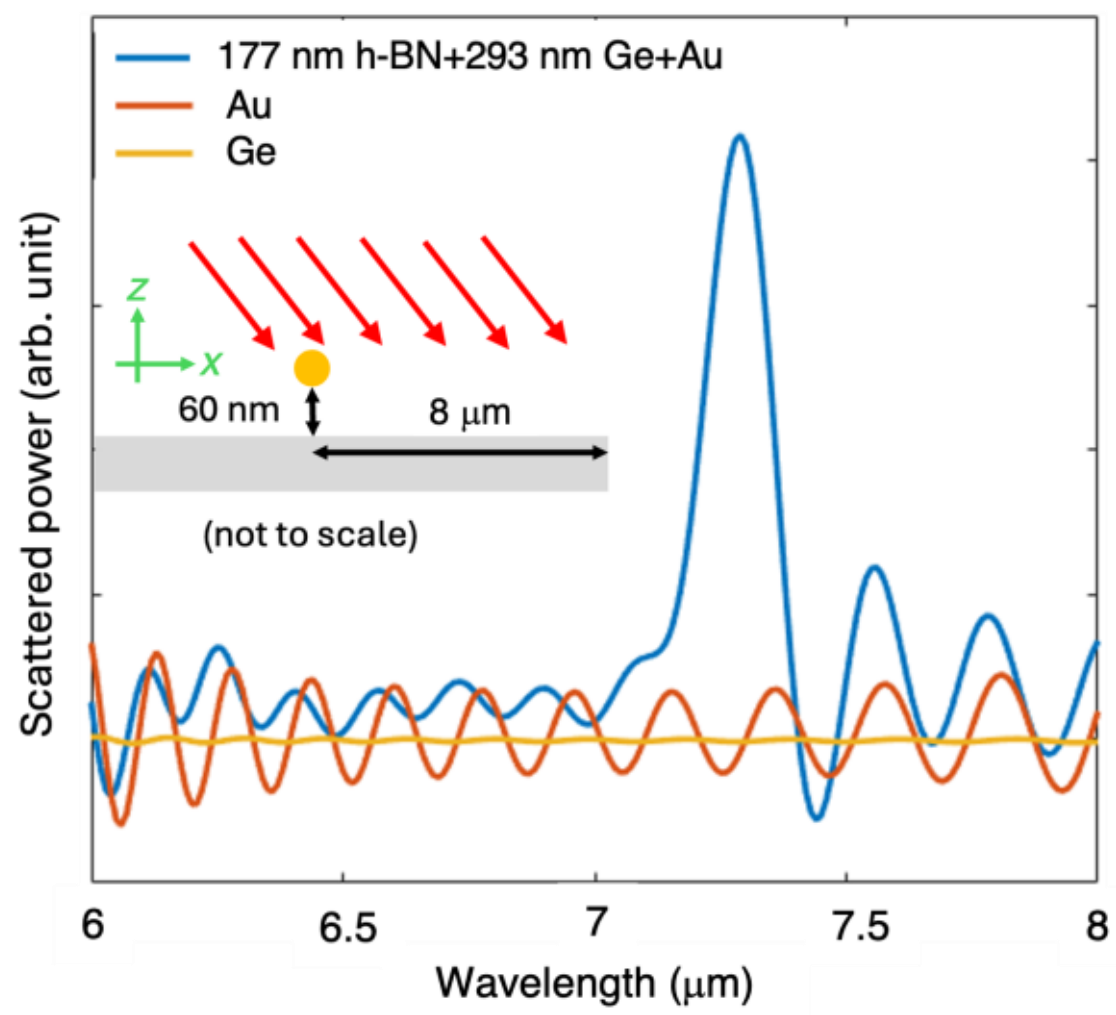

Figure S6 | Scattered power of a metallic nanosphere above the proposed heterostructure (blue curve), gold substrate (orange curve), and germanium substrate (yellow curve). The inset diagram illustrates the simulation setup, where a plane-wave background illuminates on the nanosphere placed 60 nm above the samples and 8 μm away from the sample edge. The incident angle is set to be 57 degrees - the same as the one used in the simulations of fringes presented in the main text.

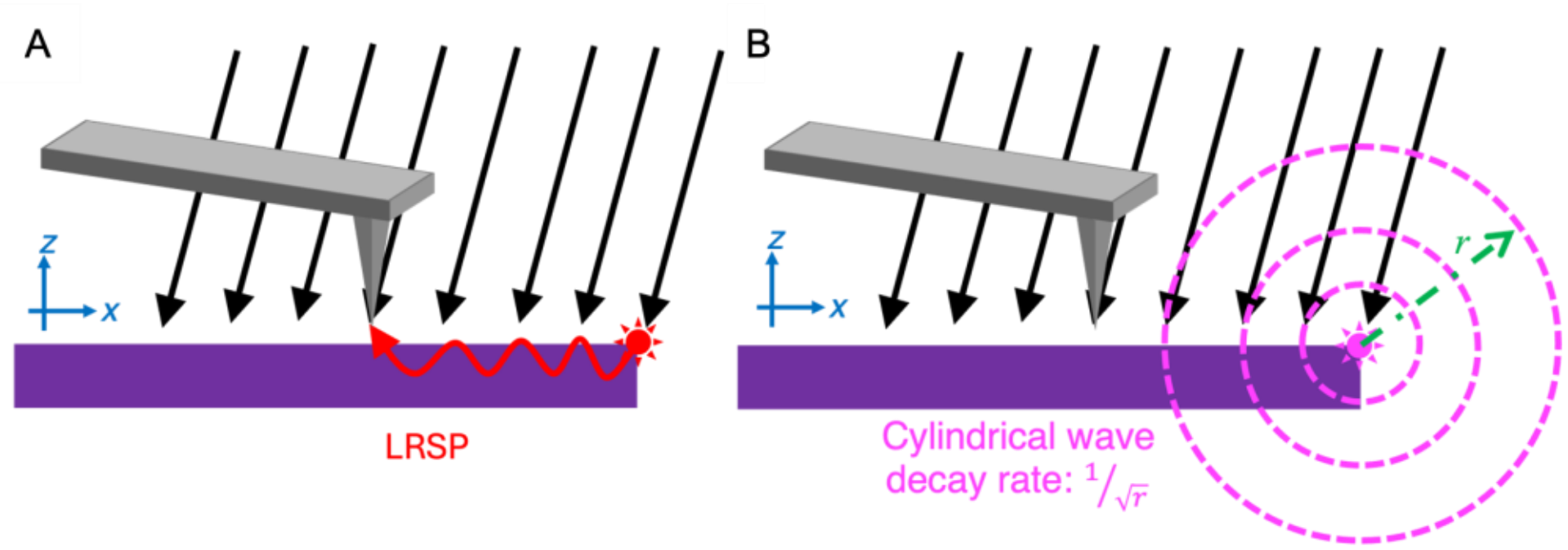

Figure S7 | Schematics of s-SNOM measurements. (**A**) LRSP and (**B**) cylindrical wave launched by the h-BN flake edge.

In addition, the propagation of cylindrical waves is independent of the optical properties of the sample*(49)*. Therefore, near-field amplitude fringes arising from these free-space/cylindrical waves are expected to exhibit similar propagation decay rates across all frequencies. However, as illustrated by the near-field amplitude image obtained at 1299 cm^{-1} in Fig. S8, near-field fringes are much weaker and decay significantly faster than the ones at near the TO phonon resonance (1370 cm^{-1}), as shown in Fig. 2 of the main text. This further rules out the possibility that the near-field amplitude fringe patterns in Fig. 2 originate from cylindrical waves launched at the h-BN flake edge.

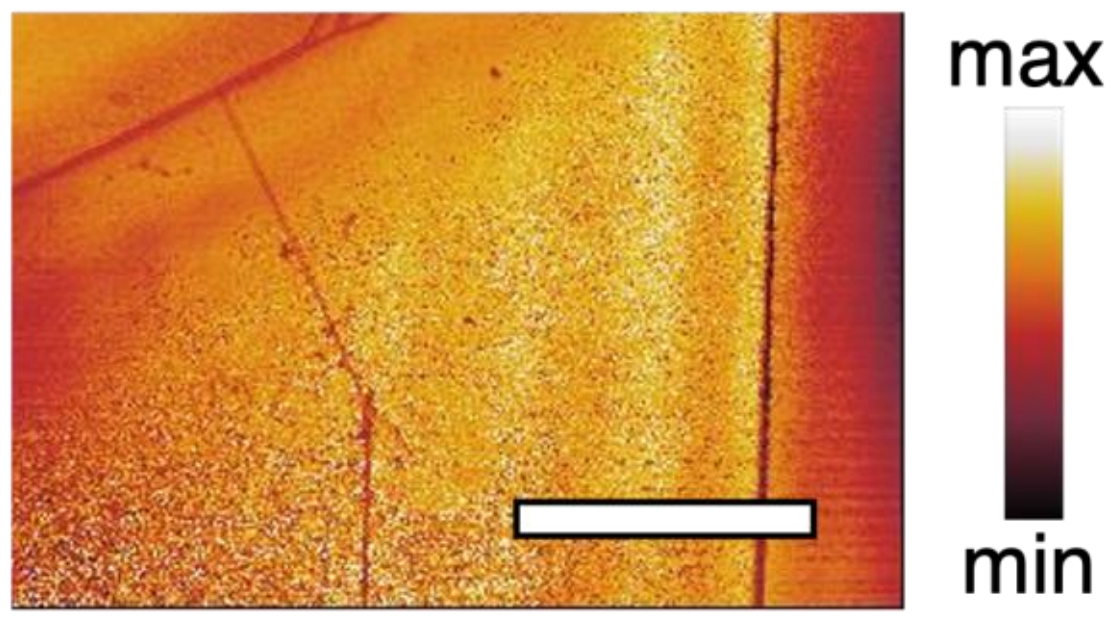

Figure S8 | Real-space near-field images of the sample in Figure S2 at 1299 cm^{-1}. The color bar is 10μm.

V. Interference model and Fourier transform of the near-field images

In the s-SNOM reflection mode measurement, the continuous wave incidence would interfere with LRSPs launched by the edge of the stratified medium. As shown in Fig. S9, an incident wave (red) impinges on the edge of the medium from the upper space, forming an incident angle θ, and an azimuthal angle ϕ with respect to the edge. The normal component (with respect to the edge) of the in-plane momentum of the incident wave is

$$k_{t,inc} = k_0 \sin\theta \sin\phi. \tag{S8}$$

The in-plane momentum of a LRSP can be written as

$$k_t = \frac{2\pi}{\lambda_t}, \tag{S9}$$

where λ_t is the in-plane wavelength of the LRSP. The interference of the two waves would form a fringe whose equal-amplitude plane is parallel to the edge. The effective momentum of the fringe then reads

$$q = k_t + k_{t,inc}. \tag{S10}$$

The peak-to-peak distance d of the fringe can be derived from $q = \frac{2\pi}{d}$, which reads $d = \frac{1}{\frac{1}{\lambda_t}+\frac{\sin\theta\sin\phi}{\lambda_0}}$, where λ_0 is the wavelength of the incident wave in free space.

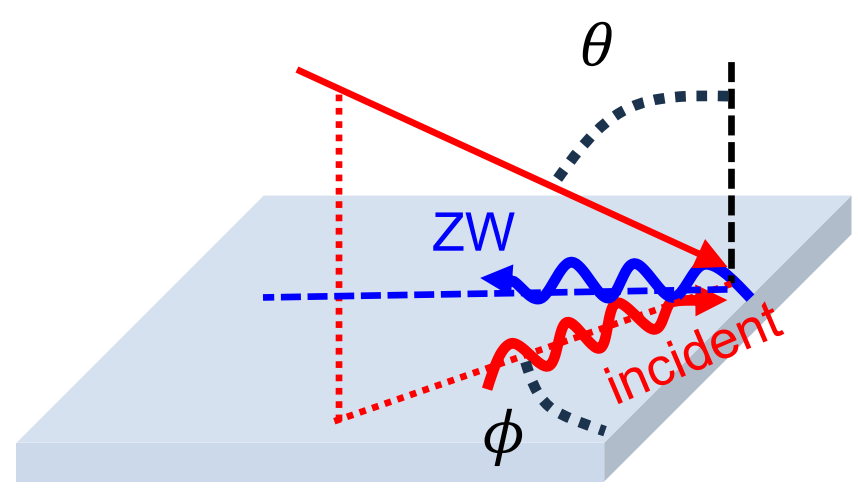

Figure S9 | Schematic of the incident angle and azimuthal angle with respect to the edge of the h-BN flake.

The raw data of the near-field images at different frequencies around the phonon resonance of the h-BN are displayed in the leftmost insets of every panel in Fig. S10. As bounded by the green rectangles, we first choose a window of images that contain noticeable fringes as our data of interest. To smooth any random noise from the imperfection of the sample and measurement, we then take the average of the pixel values along the direction of the vertical edge of the h-BN flake, which is approximately parallel to the vertical direction of the images. The average S_3 signals are shown by the orange curves in the middle insets of every panel. By fitting these curves with the third-order polynomials*(47)*, we can sufficiently estimate the background signals in the real space, which includes the non-zero bias from the interference of the LRSPs and the incident wave, as well as the interference due to the finite sizes of the h-BN flakes. After subtracting these backgrounds (black dashed curves), we obtain the pure interference patterns (without the low-frequency components) induced by the edge-launched LRSPs and the incident wave, as shown by the blue curves. Finally, the spatial Fourier transform of the pure interference patterns are depicted in the rightmost insets in Fig. S10.

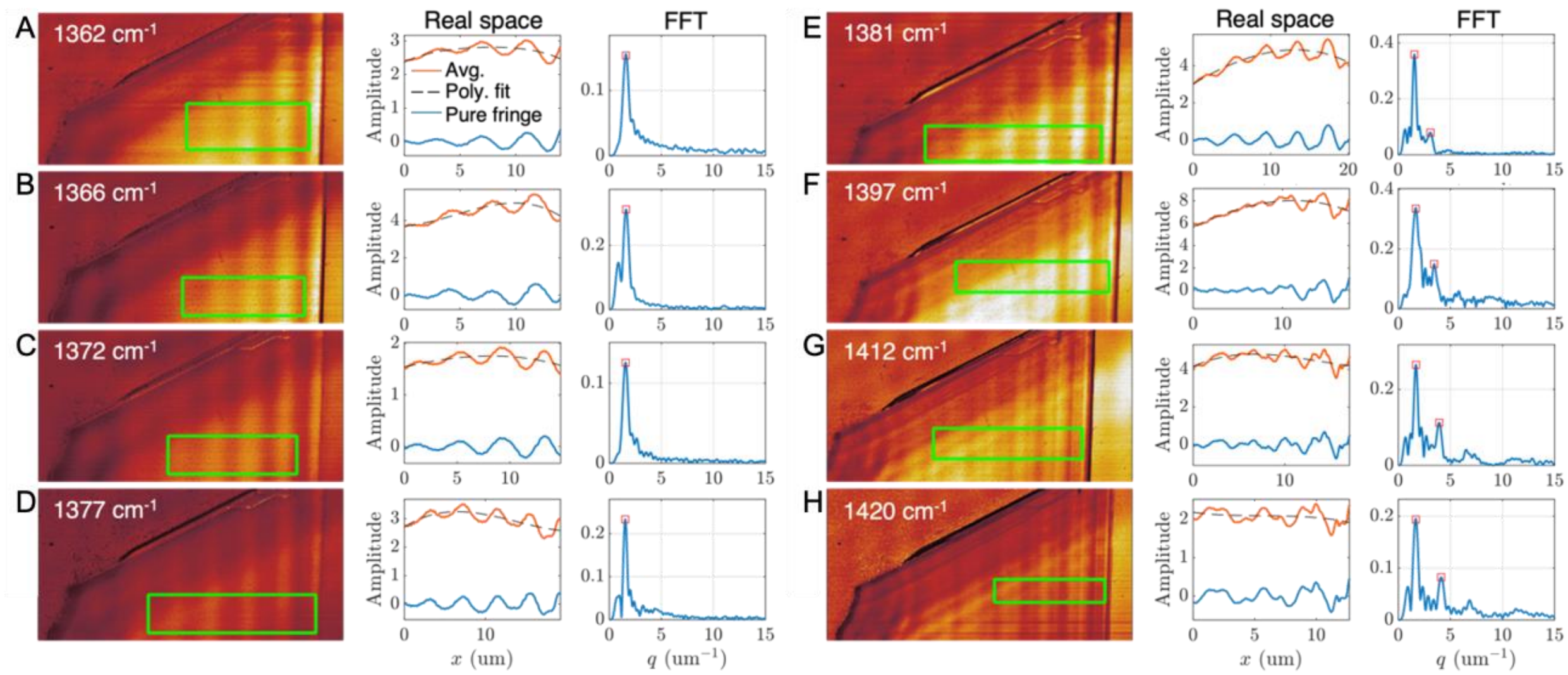

Figure S10 | Fourier transform of the near-field images.

The prominent peaks (red squares) of the Fourier spectra indicate the effective momentum of the fringes in the green boxes. We can then readily retrieve the momenta of the LRSPs using Eq. S10. The retrieved values of k_t from the peaks Figs. S10A-D and from the first peaks in Supplementary Figs. 10e-h give the eight experimental data points as shown in Fig. S1C in the main text. We further notice that there are two prominent peaks in Supplementary Figs. 10e-h. As discussed in the main text, these secondary peaks correspond to the HPhPs of a higher order, since they have almost twice the momenta of LRSPs. To verify their existence, we plot the reflection spectra with momentum k_t up to 4×10^4 cm^{-1}, as shown in Fig. S11. The retrieved momenta at eight different frequencies in Fig. S11 are overlayed on top of it. The momenta corresponding to the secondary peaks we observed in Supplementary Figs. 10 e-h indeed match with the dispersion of the higher-order mode of HPhPs, as depicted by the four blue squares ranging from $k_t = 2\sim3$ cm^{-1} and above $\omega = 1380$ cm^{-1}.

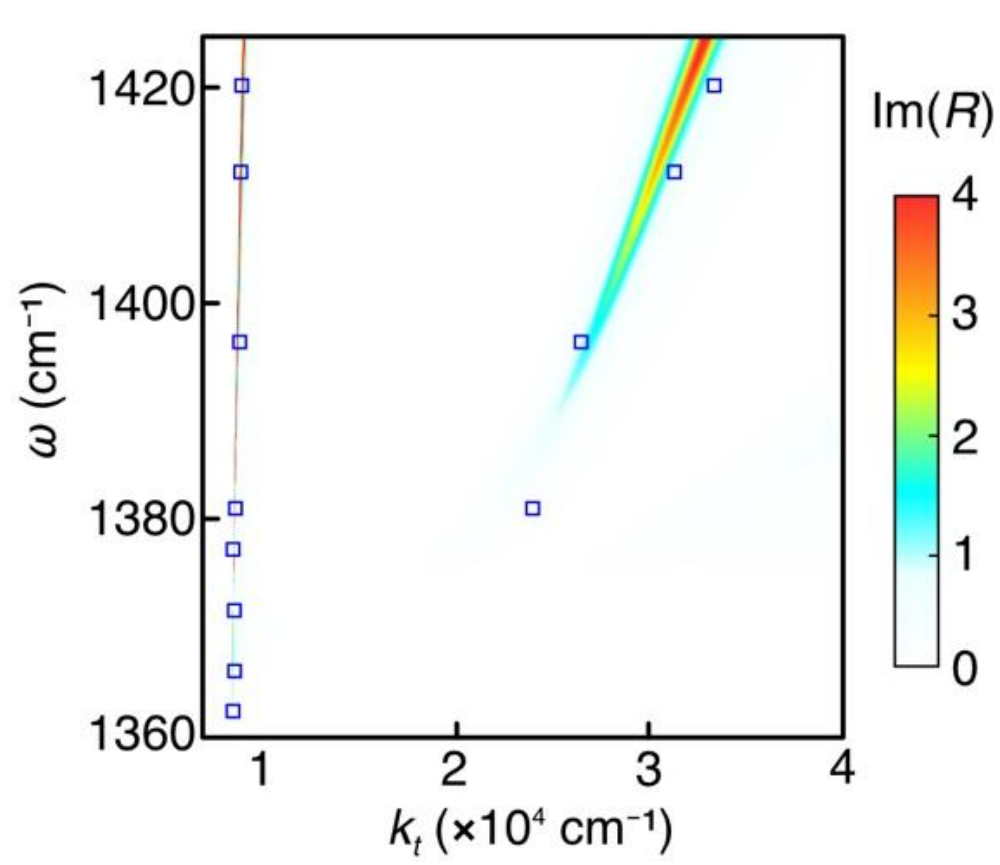

Figure S11 | Dispersion of LRSPs and higher-order HPhPs.

VI. Fig. S12-15

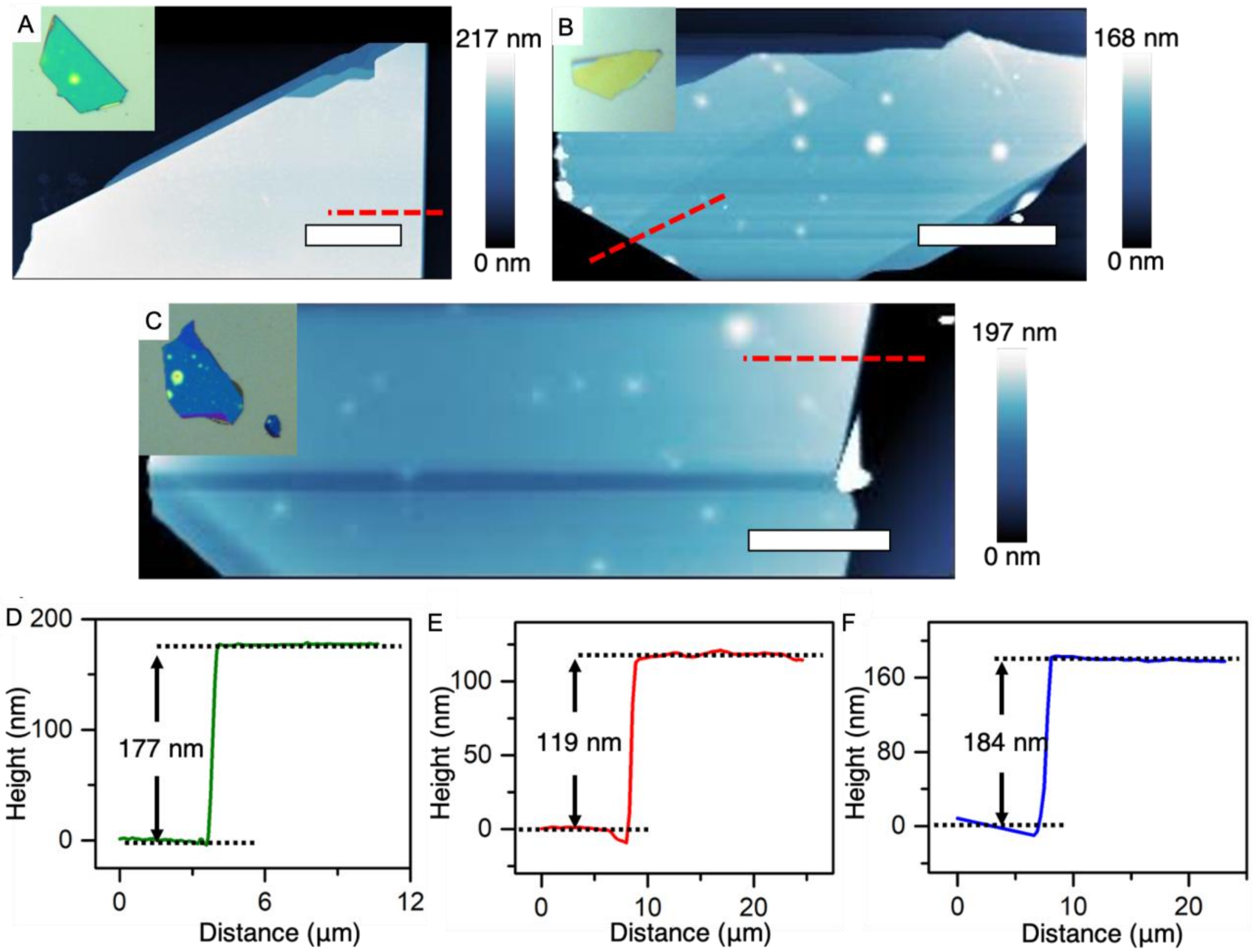

Figure S12 | AFM topography and microscopic images (inserts) of h-BN flakes. (**A-C**) The images of h-BN flakes of 177 nm h-BN+293 nm Ge sample sample (A), 119 nm h-BN+293 nm Ge sample (B), and 184 nm hBN+293 nm Ge sample (C). The scale bars are 10 (A), 22.5 (B), and 15 µm (C). (D-F) The height profiles of h-BN flakes, taken along the red dashed line (from the outside to the flake) in (A), (B), and (C), respectively.

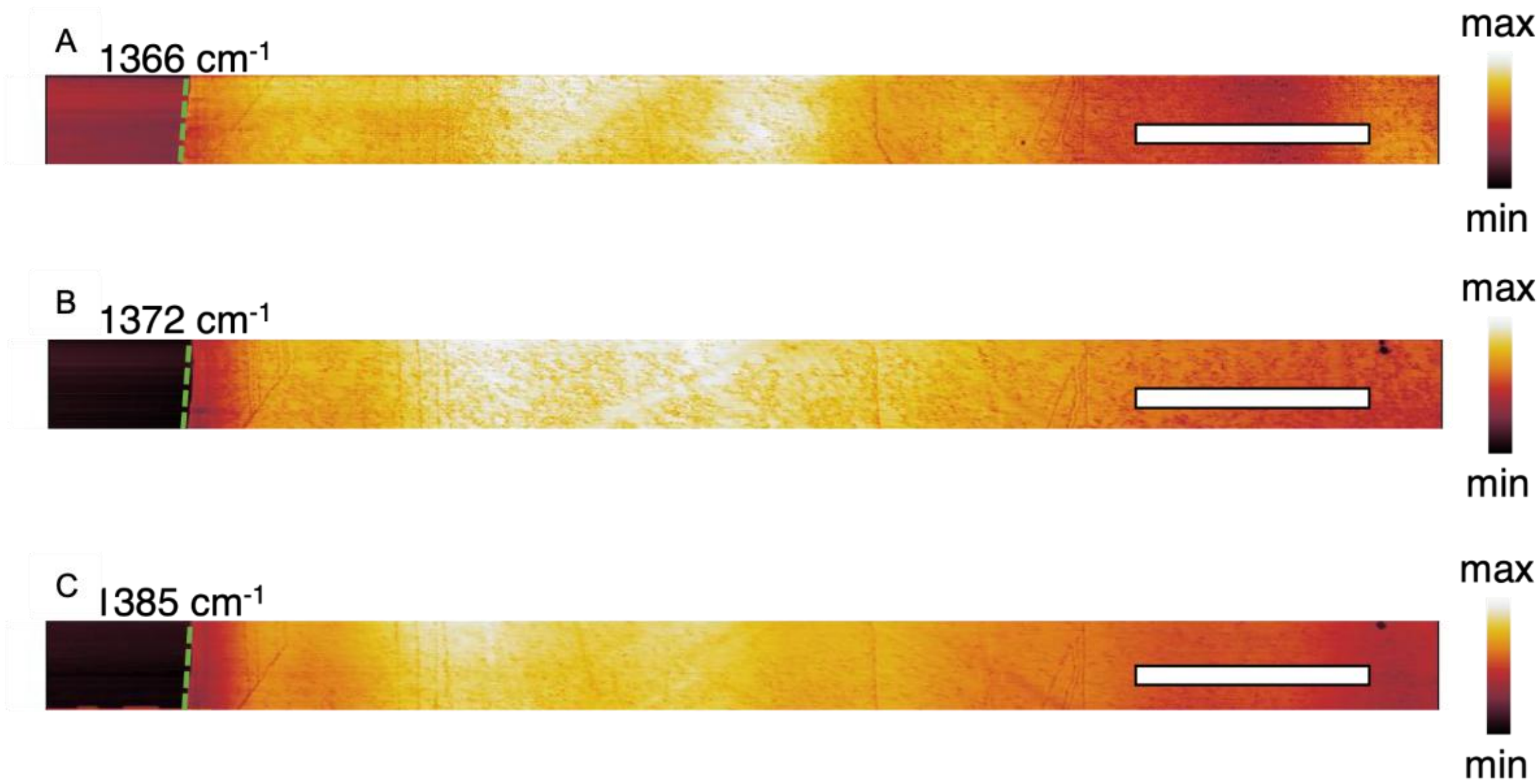

Figure S13 | Real-space near-field images of a bare h-BN flake on SiO_2 (300 nm thickness)+Si wafer. Images in (**A**), (**B**), and (**C**) are at 1366 cm^{-1}, 1372 cm^{-1}, and 1385 cm^{-1}, respectively. The azimuth angle of the incident beam to the right edge of the h-BN flake is ~90º. The dashed green line labels the edge of the h-BN flake. The thickness of h-BN is 45 nm. The scale bars are 5 μm.

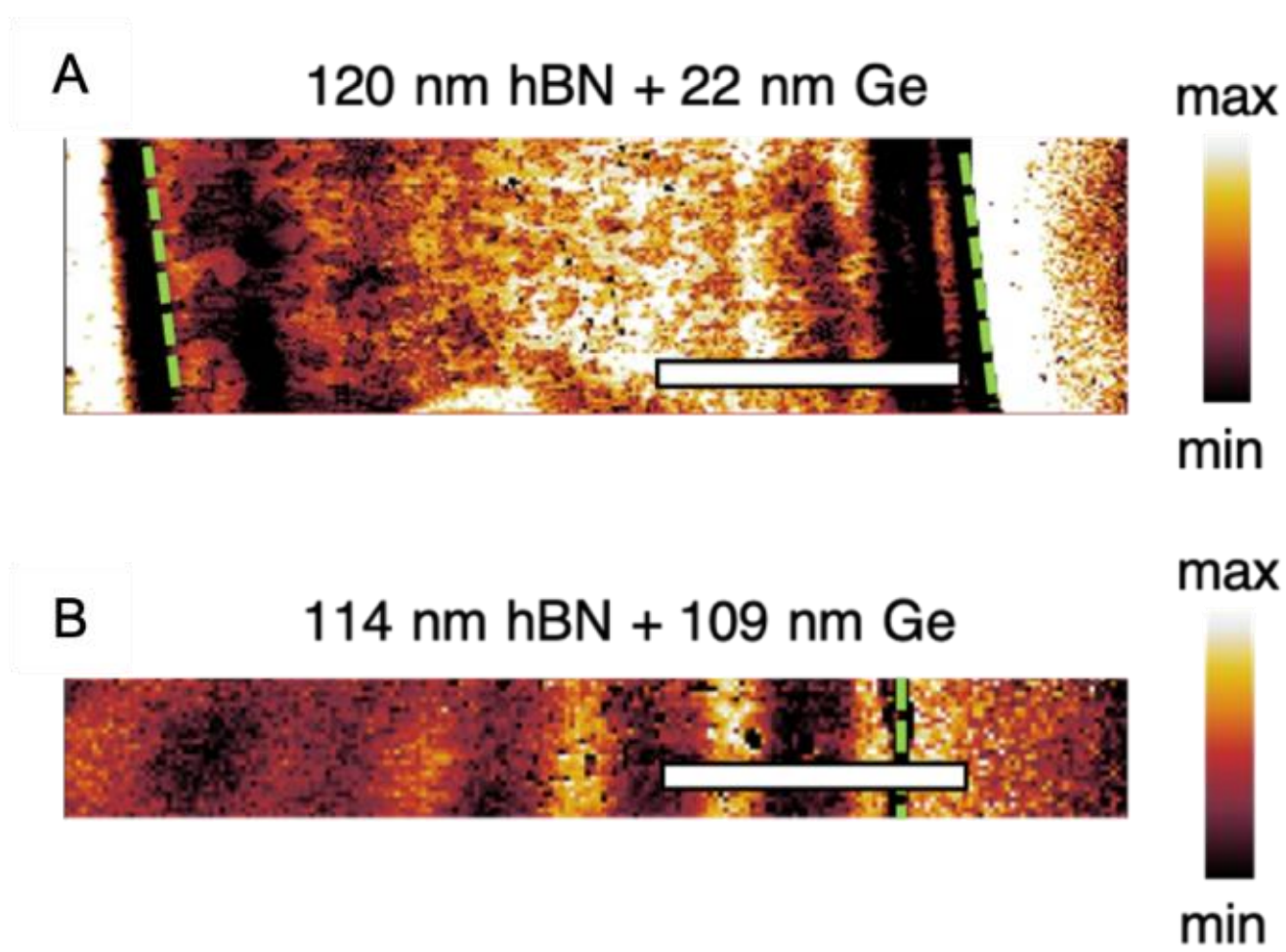

Figure S14 | Real-space near-field images of h-BN+Ge samples at 1372 cm^{-1}. The samples consist of (**A**) 120 nm h-BN and 22 nm Ge and (**B**) 114 nm h-BN and 109 Ge. The azimuth angle of the incident beam to the right edge of the h-BN flake is ~90º. The dashed green line labels the edge of the h-BN flake. The scale bars are 10 μm.

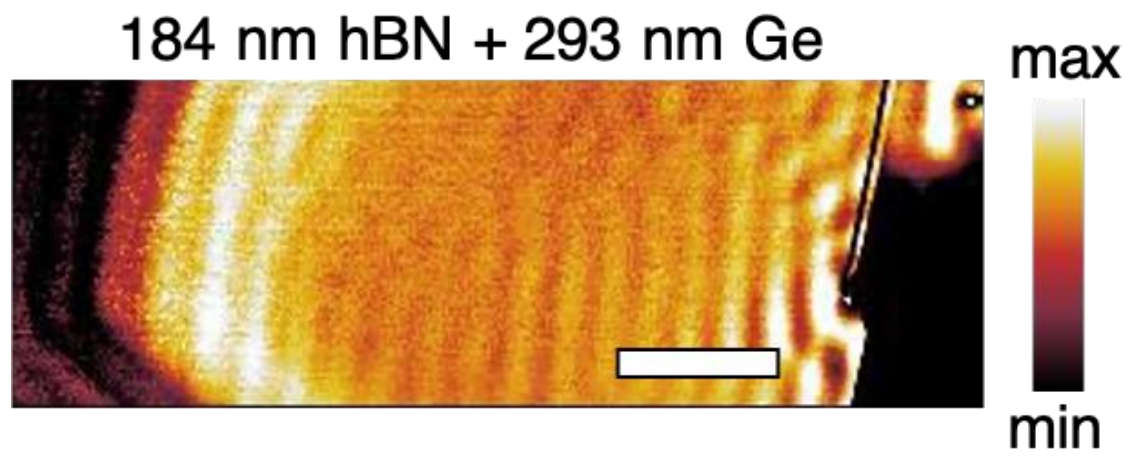

Figure S15 | Real-space near-field images of ZSWs at 1372 cm^{-1}. The sample consists of 184 nm h-BN and 293 nm Ge. The AFM and optical images of this h-BN flake are displayed in Fig. S12C. The scale bar is 15 μm.

REFERENCES

1. H. Lin, Z. Luo, T. Gu, L. C. Kimerling, K. Wada, A. Agarwal, J. Hu, Mid-infrared integrated photonics on silicon: A perspective. *Nanophotonics* **7**, 393–420 (2018).

2. D. Chen, S. D. March, A. H. Jones, Y. Shen, A. A. Dadey, K. Sun, J. A. McArthur, A. M. Skipper, X. Xue, B. Guo, J. Bai, S. R. Bank, J. C. Campbell, Photon-trapping-enhanced avalanche photodiodes for mid-infrared applications. *Nat. Photonics* **17**, 594–600 (2023).

3. B. R. Oppenheimer, S. Hinkley, High-contrast observations in optical and infrared astronomy. *Annu. Rev. Astron. Astrophys.* **47**, 253–289 (2009).

4. G. Ishigane, K. Toda, M. Tamamitsu, H. Shimada, V. R. Badarla, T. Ideguchi, Label-free mid-infrared photothermal live-cell imaging beyond video rate. *Light Sci. Appl.* **12**, 174 (2023).

5. J. Goldstein, H. Lin, S. Deckoff-Jones, M. Hempel, A.-Y. Lu, K. A. Richardson, T. Palacios, J. Kong, J. Hu, D. Englund, Waveguide-integrated mid-infrared photodetection using graphene on a scalable chalcogenide glass platform. *Nat. Commun.* **13**, 3915 (2022).

6. J. Hodgkinson, R. P. Tatam, Optical gas sensing: A review. *Meas. Sci. Technol.* **24**, 012004 (2012).

7. J.-M. Fedeli, S. Nicoletti, Mid-infrared (Mid-IR) silicon-based photonics. *Proc. IEEE* **106**, 2302–2312 (2018).

8. K. V. Voronin, G. Álvarez-Pérez, C. Lanza, P. Alonso-González, A. Y. Nikitin, Fundamentals of polaritons in strongly anisotropic thin crystal layers. *ACS Photonics* **11**, 550–560 (2024).

9. P. Díaz-Núñez, C. Lanza, Z. Wang, V. G. Kravets, J. Duan, J. Álvarez-Cuervo, A. T. Martín-Luengo, A. N. Grigorenko, Q. Yang, A. Paarmann, J. Caldwell, P. Alonso-González, A. Mishchenko, Visualization of topological shear polaritons in gypsum thin films. *Sci. Adv.* **11**, eadw3452 (2025).

10. D. N. Basov, M. M. Fogler, F. J. García de Abajo, Polaritons in van der Waals materials. *Science* **354**, aag1992 (2016).

11. G. Carini, M. Pradhan, E. Gelžinytė, A. Ardenghi, S. Dixit, M. Obst, A. S. Senarath, N. S. Mueller, G. Álvarez-Pérez, K. Diaz-Granados, R. A. Kowalski, R. Niemann, F. G. Kaps, J. Wetzel, R. B. Iyer, P. Mazzolini, M. Schubert, J. M. Klopf, J. T. Margraf, O. Bierwagen, M. Wolf, K. Reuter, L. M. Eng, S. C. Kehr, J. D. Caldwell, C. Carbogno, T. G. Folland, M. R. Wagner, A. Paarmann, Spectral tuning of hyperbolic shear polaritons in monoclinic gallium oxide via isotopic substitution. *Adv. Mater.* **38**, e14561 (2026).

12. N. C. Passler, X. Ni, G. Hu, J. R. Matson, G. Carini, M. Wolf, M. Schubert, A. Alù, J. D. Caldwell, T. G. Folland, A. Paarmann, Hyperbolic shear polaritons in low-symmetry crystals. *Nature* **602**, 595–600 (2022).

13. J. Matson, S. Wasserroth, X. Ni, M. Obst, K. Diaz-Granados, G. Carini, E. M. Renzi, E. Galiffi, T. G. Folland, L. M. Eng, J. Michael Klopf, S. Mastel, S. Armster, V. Gambin, M. Wolf, S. C. Kehr, A. Alù, A. Paarmann, J. D. Caldwell, Controlling the propagation asymmetry of hyperbolic shear polaritons in beta-gallium oxide. *Nat. Commun.* **14**, 5240 (2023).

14. L. Zhou, X. Ni, Z. Wang, E. M. Renzi, J. Xu, Z. Zhou, Y. Yin, Y. Yin, R. Song, Z. Zhao, K. Yu, D. Huang, Z. Wang, X. Cheng, A. Alù, T. Jiang, Engineering shear polaritons in 2D twisted heterostructures. *Nat. Commun.* **16**, 2953 (2025).

15. Z. Zheng, F. Sun, W. Huang, J. Jiang, R. Zhan, Y. Ke, H. Chen, S. Deng, Phonon polaritons in twisted double-layers of hyperbolic van der Waals crystals. *Nano Lett.* **20**, 5301–5308 (2020).

16. G. Hu, W. Ma, D. Hu, J. Wu, C. Zheng, K. Liu, X. Zhang, X. Ni, J. Chen, X. Zhang, Q. Dai, J. D. Caldwell, A. Paarmann, A. Alù, P. Li, C.-W. Qiu, Real-space nanoimaging of hyperbolic shear polaritons in a monoclinic crystal. *Nat. Nanotechnol.* **18**, 64–70 (2023).

17. M. Suriyage, Q. Zhou, H. Qin, X. Sun, Z. Lu, S. A. Maier, Z. Yu, Y. Lu, Long-propagating ghost phonon polaritons enabled by selective mode excitation. *Light Sci. Appl.* **14**, 254 (2025).

18. A. I. F. Tresguerres-Mata, O. G. Matveeva, C. Lanza, J. Álvarez-Cuervo, K. V. Voronin, F. Calavalle, G. Avedissian, P. Díaz-Núñez, G. Álvarez-Pérez, A. Tarazaga Martín-Luengo, J.

Taboada-Gutiérrez, J. Duan, J. Martín-Sánchez, A. Bylinkin, R. Hillenbrand, A. Mishchenko, L. E. Hueso, V. S. Volkov, A. Y. Nikitin, P. Alonso-González, Directional strong coupling at the nanoscale between hyperbolic polaritons and organic molecules. *Nat. Photonics* **19**, 1196–1202 (2025).

19. Q. Guo, R. Yu, C. Li, S. Yuan, B. Deng, F. J. García de Abajo, F. Xia, Efficient electrical detection of mid-infrared graphene plasmons at room temperature. *Nat. Mater.* **17**, 986–992 (2018).

20. F. H. L. Koppens, D. E. Chang, F. J. García de Abajo, Graphene plasmonics: A platform for strong light–matter interactions. *Nano Lett.* **11**, 3370–3377 (2011).

21. Z. Fei, A. S. Rodin, G. O. Andreev, W. Bao, A. S. McLeod, M. Wagner, L. M. Zhang, Z. Zhao, M. Thiemens, G. Dominguez, M. M. Fogler, A. H. C. Neto, C. N. Lau, F. Keilmann, D. N. Basov, Gate-tuning of graphene plasmons revealed by infrared nano-imaging. *Nature* **487**, 82–85 (2012).

22. J. Chen, M. Badioli, P. Alonso-González, S. Thongrattanasiri, F. Huth, J. Osmond, M. Spasenović, A. Centeno, A. Pesquera, P. Godignon, A. Zurutuza Elorza, N. Camara, F. J. G. de Abajo, R. Hillenbrand, F. H. L. Koppens, Optical nano-imaging of gate-tunable graphene plasmons. *Nature* **487**, 77–81 (2012).

23. Y. Dong, L. Xiong, I. Y. Phinney, Z. Sun, R. Jing, A. S. McLeod, S. Zhang, S. Liu, F. L. Ruta, H. Gao, Z. Dong, R. Pan, J. H. Edgar, P. Jarillo-Herrero, L. S. Levitov, A. J. Millis, M. M. Fogler, D. A. Bandurin, D. N. Basov, Fizeau drag in graphene plasmonics. *Nature* **594**, 513–516 (2021).

24. H. Hu, R. Yu, H. Teng, D. Hu, N. Chen, Y. Qu, X. Yang, X. Chen, A. S. McLeod, P. Alonso-González, X. Guo, C. Li, Z. Yao, Z. Li, J. Chen, Z. Sun, M. Liu, F. J. García de Abajo, Q. Dai, Active control of micrometer plasmon propagation in suspended graphene. *Nat. Commun.* **13**, 1465 (2022).

25. A. N. Grigorenko, M. Polini, K. S. Novoselov, Graphene plasmonics. *Nat. Photonics* **6**, 749–758 (2012).

26. L. Ju, B. Geng, J. Horng, C. Girit, M. Martin, Z. Hao, H. A. Bechtel, X. Liang, A. Zettl, Y. R. Shen, F. Wang, Graphene plasmonics for tunable terahertz metamaterials. *Nat. Nanotechnol.* **6**, 630–634 (2011).

27. T. Low, A. Chaves, J. D. Caldwell, A. Kumar, N. X. Fang, P. Avouris, T. F. Heinz, F. Guinea, L. Martin-Moreno, F. Koppens, Polaritons in layered two-dimensional materials. *Nat. Mater.* **16**, 182–194 (2017).

28. M. Wang, G. Hu, S. Chand, M. Cotrufo, Y. Abate, K. Watanabe, T. Taniguchi, G. Grosso, C.-W. Qiu, A. Alù, Spin-orbit-locked hyperbolic polariton vortices carrying reconfigurable topological charges. *eLight* **2**, 12 (2022).

29. S. Dai, Z. Fei, Q. Ma, A. S. Rodin, M. Wagner, A. S. McLeod, M. K. Liu, W. Gannett, W. Regan, K. Watanabe, T. Taniguchi, M. Thiemens, G. Dominguez, A. H. C. Neto, A. Zettl, F. Keilmann, P. Jarillo-Herrero, M. M. Fogler, D. N. Basov, Tunable phonon polaritons in atomically thin van der Waals crystals of boron nitride. *Science* **343**, 1125–1129 (2014).

30. G. Hu, Q. Ou, G. Si, Y. Wu, J. Wu, Z. Dai, A. Krasnok, Y. Mazor, Q. Zhang, Q. Bao, C.-W. Qiu, A. Alù, Topological polaritons and photonic magic angles in twisted α-MoO_3 bilayers. *Nature* **582**, 209–213 (2020).

31. W. Ma, P. Alonso-González, S. Li, A. Y. Nikitin, J. Yuan, J. Martín-Sánchez, J. Taboada-Gutiérrez, I. Amenabar, P. Li, S. Vélez, C. Tollan, Z. Dai, Y. Zhang, S. Sriram, K. Kalantar-Zadeh, S.-T. Lee, R. Hillenbrand, Q. Bao, In-plane anisotropic and ultra-low-loss polaritons in a natural van der Waals crystal. *Nature* **562**, 557–562 (2018).

32. P. Li, I. Dolado, F. J. Alfaro-Mozaz, F. Casanova, L. E. Hueso, S. Liu, J. H. Edgar, A. Y. Nikitin, S. Vélez, R. Hillenbrand, Infrared hyperbolic metasurface based on nanostructured van der Waals materials. *Science* **359**, 892–896 (2018).

33. H. Hu, N. Chen, H. Teng, R. Yu, M. Xue, K. Chen, Y. Xiao, Y. Qu, D. Hu, J. Chen, Z. Sun, P. Li, F. J. G. de Abajo, Q. Dai, Gate-tunable negative refraction of mid-infrared polaritons. *Science* **379**, 558–561 (2023).

34. A. J. Sternbach, S. L. Moore, A. Rikhter, S. Zhang, R. Jing, Y. Shao, B. S. Y. Kim, S. Xu, S. Liu, J. H. Edgar, A. Rubio, C. Dean, J. Hone, M. M. Fogler, D. N. Basov, Negative refraction in hyperbolic hetero-bicrystals. *Science* **379**, 555–557 (2023).

35. W. Ma, G. Hu, D. Hu, R. Chen, T. Sun, X. Zhang, Q. Dai, Y. Zeng, A. Alù, C.-W. Qiu, P. Li, Ghost hyperbolic surface polaritons in bulk anisotropic crystals. *Nature* **596**, 362–366 (2021).

36. G. X. Ni, A. S. McLeod, Z. Sun, L. Wang, L. Xiong, K. W. Post, S. S. Sunku, B.-Y. Jiang, J. Hone, C. R. Dean, M. M. Fogler, D. N. Basov, Fundamental limits to graphene plasmonics. *Nature* **557**, 530–533 (2018).

37. X. Ni, G. Carini, W. Ma, E. M. Renzi, E. Galiffi, S. Wasserroth, M. Wolf, P. Li, A. Paarmann, A. Alù, Observation of directional leaky polaritons at anisotropic crystal interfaces. *Nat. Commun.* **14**, 2845 (2023).

38. A. J. Giles, S. Dai, I. Vurgaftman, T. Hoffman, S. Liu, L. Lindsay, C. T. Ellis, N. Assefa, I. Chatzakis, T. L. Reinecke, J. G. Tischler, M. M. Fogler, J. H. Edgar, D. N. Basov, J. D. Caldwell, Ultralow-loss polaritons in isotopically pure boron nitride. *Nat. Mater.* **17**, 134–139 (2018).

39. J. S. Fakonas, H. Lee, Y. A. Kelaita, H. A. Atwater, Two-plasmon quantum interference. *Nat. Photonics* **8**, 317–320 (2014).

40. W. C. Chew, *Waves and Fields in Inhomogenous Media* (John Wiley & Sons, 1999).

41. V. M. Agranovich, D. L. Mills, *Surface Polaritons* (North-Holland, Amsterdam, 1982).

42. S. Dai, W. Fang, N. Rivera, Y. Stehle, B.-Y. Jiang, J. Shen, R. Y. Tay, C. J. Ciccarino, Q. Ma, D. Rodan-Legrain, P. Jarillo-Herrero, E. H. T. Teo, M. M. Fogler, P. Narang, J. Kong, D. N. Basov, Phonon polaritons in monolayers of hexagonal boron nitride. *Adv. Mater.* **31**, e1806603 (2019).

43. S. Dai, Q. Ma, T. Andersen, A. S. Mcleod, Z. Fei, M. K. Liu, M. Wagner, K. Watanabe, T. Taniguchi, M. Thiemens, F. Keilmann, P. Jarillo-Herrero, M. M. Fogler, D. N. Basov,

Subdiffractional focusing and guiding of polaritonic rays in a natural hyperbolic material. *Nat. Commun.* **6**, 6963 (2015).

44. G. Tsandoulas, Excitation of a grounded dielectric slab by a horizontal dipole. *IEEE Trans. Antennas Propag.* **17**, 156–161 (1969).

45. D. Sarid, Long-range surface-plasma waves on very thin metal films. *Phys. Rev. Lett.* **47**, 1927–1930 (1981).

46. P. Berini, Long-range surface plasmon polaritons. *Adv. Opt. Photonics* **1**, 484–588 (2009).

47. S. G. Menabde, S. Boroviks, J. Ahn, J. T. Heiden, K. Watanabe, T. Taniguchi, T. Low, D. K. Hwang, N. A. Mortensen, M. S. Jang, Near-field probing of image phonon-polaritons in hexagonal boron nitride on gold crystals. *Sci. Adv.* **8**, eabn0627 (2022).

48. M. Chen, Y. Zhong, E. Harris, J. Li, Z. Zheng, H. Chen, J.-S. Wu, P. Jarillo-Herrero, Q. Ma, J. H. Edgar, X. Lin, S. Dai, Van der Waals isotope heterostructures for engineering phonon polariton dispersions. *Nat. Commun.* **14**, 4782 (2023).

49. V. E. Babicheva, S. Gamage, L. Zhen, S. B. Cronin, V. S. Yakovlev, Y. Abate, Near-field surface waves in few-layer MoS2. *ACS Photonics* **5**, 2106–2112 (2018).

50. K. Y. Ma, M. Kim, H. S. Shin, Large-area hexagonal boron nitride layers by chemical vapor deposition: Growth and applications for substrates, encapsulation, and membranes. *Acc. Mater. Res.* **3**, 748–760 (2022).

51. A. S. McKeown-Green, H. J. Zeng, A. P. Saunders, J. Li, J. Shi, Y. Shen, F. Pan, J. Hu, J. A. Dionne, T. F. Heinz, S. M. Wu, F. Zheng, F. Liu, Millimeter-scale exfoliation of hBN with tunable flake thickness for scalable encapsulation. *ACS Appl. Nano Mater.* **7**, 6574–6582 (2024).

52. A. S. McLeod, P. Kelly, M. D. Goldflam, Z. Gainsforth, A. J. Westphal, G. Dominguez, M. H. Thiemens, M. M. Fogler, D. N. Basov, Model for quantitative tip-enhanced spectroscopy and the extraction of nanoscale-resolved optical constants. *Phys. Rev. B* **90**, 085136 (2014).

53. K. Sarkar, M. N. Abdallah, M. Salazar-Palma, W. M. Dyab, Surface plasmons-polaritons, surface waves, and Zenneck waves: Clarification of the terms and a description of the concepts and their evolution. *IEEE Antennas Propag. Mag.* **59**, 77–93 (2017).
